# A Highly Selective and Sensitive Sequential Recognition Probe Zn^2+^ and H_2_PO_4_^−^ Based on Chiral Thiourea Schiff Base

**DOI:** 10.3390/molecules28104166

**Published:** 2023-05-18

**Authors:** Shan Yang, Yichuan Huang, Aidang Lu, Ziwen Wang, Hongyan Li

**Affiliations:** 1 School of Chemical Engineering and Technology, Hebei University of Technology, Tianjin 300401, China; 17865513875@163.com (S.Y.); yichuan1026@163.com (Y.H.); 2 Tianjin Key Laboratory of Structure and Performance for Functional Molecules, College of Chemistry, Tianjin Normal University, Tianjin 300387, China

**Keywords:** chiral thiourea, Schiff base, fluoresce, Zn^2+^ detection, H_2_PO_4_^−^ detection

## Abstract

A series of novel chiral thiourea fluorescent probes **HL_1_**–**HL_6_** were designed and synthesized from (1*R*,2*R*)-1,2-diphenylethylenediamine, phenyl isothiocyanate, and different substituted salicylic aldehydes. All of the compounds were confirmed by ^1^H NMR, ^13^C NMR, and HRMS. They exhibit high selectivity and sensitivity to Zn^2+^ in the presence of nitrate ions with the detection limit of 2.3 × 10^−8^ M (**HL_5_**). Meanwhile, their zinc (II) complexes (**L**-ZnNO_3_) showed continuous response to H_2_PO_4_^−^ in acetonitrile solution. The identification processes could further be verified by supramolecular chemistry data analysis, X-ray single-crystal diffraction analysis, and theoretical study. The research provides reliable evidence for an explanation of the mechanism of action of thiourea involved in coordination, which is important for the application of thiourea fluorescent probes. In short, the sensors **HL_1_**–**HL_6_** based on chiral thiourea Schiff base will be promising detection devices for Zn^2+^ and H_2_PO_4_^−^.

## 1. Introduction

In recent years, widespread research has been undertaken on the detection of metal ions [1,2,3,4,5] and inorganic anions [6,7,8,9], particularly within the development of selective ion probes, which has more generally become a popular topic in scientific research. Zn^2+^ is the second most common element in the human body, after iron, and not only plays an important role in protein structure, catalysis, transcription, and regulation, but is also closely associated with nerve signal transmission, enzyme regulation, and gene expression [10]. Excessive concentrations of Zn^2+^ can cause neurodegenerative diseases such as Alzheimer’s disease and Parkinson’s disease [11,12,13,14]. Therefore, the development of methods to accurately determine trace amounts of Zn^2+^ and for its removal prior to entering the body are important research goals. 

Among the various anions, the detection of phosphates most commonly the subject of research. Phosphates are the key substrates of many biochemical reactions and the main components of biomolecules [15,16,17,18]. Phosphate and its derivatives are important nutrients, and the basic life activities of organisms are closely related to phosphate. Phosphate is not only a component of some important structures of plants but is also a catalyst for many key biochemical reactions in plants, which can promote the development of plant roots and the early growth of seedlings. The life activities of organisms are also closely related to phosphates [18]. Phosphates can regulate the level of 1,25-(OH) 2-vitamin D in human plasma [19] and react with hydroxyl groups to form phosphate esters, thus allowing monomers to polymerize into relatively stable long chain skeletons (DNA and RNA skeletons) [20]. In addition, phosphate is also involved in ATP energy supply, ion channel regulation, enzymatic reactions, and intercellular signal transduction [8]. Unfortunately, a large amount of phosphate deposition can lead to a lack of dissolved oxygen in water and algae eutrophication [18].

At present, there are many conventional methods to detect Zn^2+^ ions including ion chromatography, atomic absorption spectrometry, and ion exchange [21]. Among these methods, chemical sensors are widely used to detect many biological and environmental heavy metal pollutants because of their advantages such as higher sensitivity, lower cost, and faster detection [22,23]. In particular, the multi-functional sensors used for the sequential recognition of cations and anions have remarkable advantages in special applications for their low cost, quick response, and convenient operation. Most of the currently available fluorescent probes can only analyze certain types of anions or cations, and there are few fluorescent sensors with the sequential recognition of Zn^2+^ and H_2_PO_4_^−^ [24,25].

**Figure 1:**
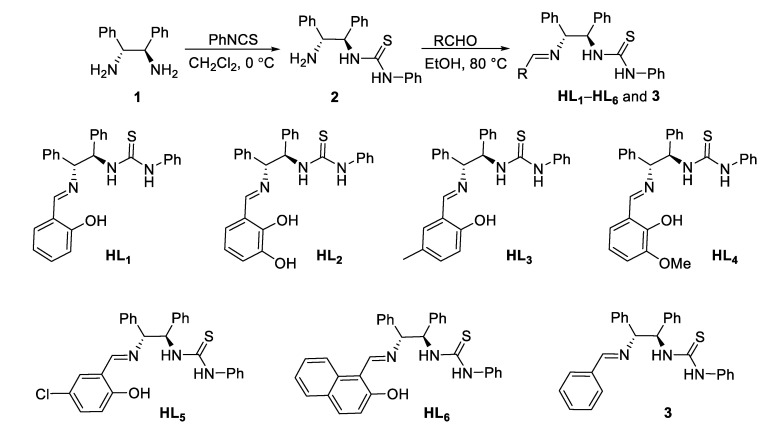
Synthesis of **HL_1_**–**HL_6_** and compound **3**.

In this work, a series of chiral thiourea Schiff base fluorescent probes (**HL_1_**–**HL_6_**) were designed and synthesized starting from (1*R*,2*R*)-1,2-diphenylethylenediamine, phenyl isothiocyanate, and different substituted salicylic aldehydes (Figure 1). They were explored for the first time for fluorescent-responsive Zn^2+^ detection in the presence of nitrate ions in acetonitrile. Meanwhile, their zinc (II) complexes (**HL**-**Zn^2+^**-**NO_3_^−^**) showed continuous response to H_2_PO_4_**^−^** in acetonitrile solution. Up to now, the detection mechanisms of thiourea probes have been reported mostly by ^1^H NMR titration or Job’s plot titration experiments to speculate the possible coordination mode, and some of the assays do not provide practical and reliable data [26,27]. Here, we successfully elucidated the mechanism of metal ion recognition by this kind of thiourea Schiff base fluorescent probe using supramolecular chemistry data analysis, X-ray single-crystal diffraction analysis, and theoretical study. The research provides reliable evidence for an explanation of the mechanism of action of thiourea involved in coordination, which is important for the application of thiourea fluorescent probes. The probes in our work have the advantages of high sensitivity and low detection limits. In short, the sensors **HL_1_**–**HL_6_** based on chiral thiourea Schiff base will be promising detection devices for Zn^2+^ and H_2_PO_4_^−^.

## 2. Results and Discussion

### 2.1. Fluorescence Spectroscopic Studies of ***HL_1_***–***HL_6_*** and ***3*** in the Presence of Metal Ions

#### 2.1.1. Cation Selectivity Experiments

**Figure 2:**
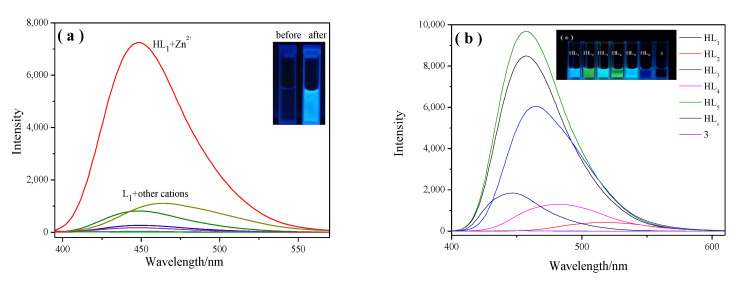
(**a**) Change in the emission spectra of **HL_1_** upon the addition of different metal ions in acetonitrile. Inset: Visual color change of **HL_1_** before and after the addition of Zn^2+^ under a 365 nm UV lamp; (**b**) fluorescence spectra of **HL_1_**–**HL_6_** and compound **3** after the addition of Zn^2+^; (**c**) color of **HL_1_**–**HL_6_** and compound **3** solution with the addition of Zn^2+^ under a UV lamp.

Selectivity experiments were carried out for **HL_1_–HL_6_** and 3 by examining the emission spectra in the presence of different metal ions (Ag^+^, Na^+^, K^+^, Co^2+^, Fe^3+^, Cu^2+^, Cd^2+^, Cr^3+^, Mg^2+^, Pb^2+^, Ni^2+^, Zn^2+^, Fe^2+^, Hg^2+^, Ca^2+^) in filtered Milli-Q water. As shown in Figure 2a, **HL_1_** displayed a weak emission peak at 447 nm in acetonitrile solution when excited at 380 nm. Upon the addition of various metal ions in **HL_1_**, the emission spectrum was only significantly enhanced with Zn^2+^, whereas Cd^2+^ and Pb^2+^ caused a very small enhancement in the emission intensity at wavelengths corresponding to 400 and 600 nm, respectively. This observation demonstrates the high selectivity of **HL_1_** towards Zn^2+^ compared to other metal ions, including Cd^2+^ and Pb^2+^. Molecular probes that show turn-on fluorescence signaling upon interaction with Zn^2+^ also often interact with Cd^2+^/Pb^2+^, resulting in turn-on signaling [28]. In contrast, blue fluorescence was observed in the probe solution upon interaction with Zn^2+^ under a 365 nm UV lamp (Figure 2a, inset). In the case of other metal cations, the fluorescence spectra almost remained unchanged (Figure 2a). Subsequently, the selectivity of the **HL_1_–HL_6_** and **3** probes for various metal ions was investigated (Figures S1 and S2). The addition of Zn^2+^ resulted in a prominent luminescence enhancement (Figures S1 and S2), whereas the addition of a large excess of other competitive cations caused only slight luminescent changes (Figure S2), with the exception of **3** (Figures S1f and S2g). The fluorescence emission produced by the interaction between **HL_5_** and Zn^2+^ was the strongest, whereas that with **HL_2_** was the weakest. The strength of the other observed emissions was at a medium level, except for compound **3**. The difference in the fluorescence intensity of other compounds mainly depends on the difference in the side substituents of salicylaldehyde. The fluorescence intensity of **HL_5_** is better than that of **HL_3_**, and obviously stronger than that of **HL_4_**, **HL_6_**, and **HL_2_**. When salicylaldehyde contains electron-donating groups, such as –OH, –CH_3_, and –OCH_3_, it can be increased the density of the electron cloud on the benzene ring, resulting in the weakening of the coordination ability of **HL** with zinc ions. Under the combined influence of the inductive effect and mesomeric effect, **HL_5_** containing –Cl has the strongest coordination ability with Zn^2+^. Compound 3 without the –OH in the benzene ring structure of the benzylidene group was not able to recognize Zn^2+^, which demonstrates that the phenolic hydroxyl group was involved in the formation of complexes. **HL_1_**–**HL_6_** probes showed high selectivity in sensing Zn^2+^ and can be used as “turn-on” luminescent molecular probes toward Zn^2+^.

#### 2.1.2. Cation-Competitive Experiments

**Figure 3:**
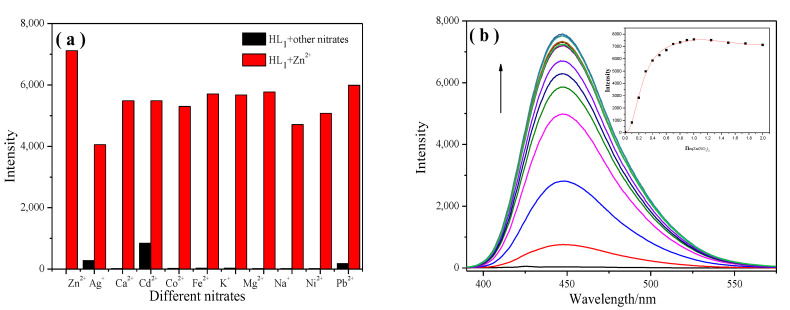
(**a**) Fluorescence intensities of **HL_1_** excited at 377 nm upon the addition of Zn(NO_3_)_2_ in the presence of nitrates subject to interference. The black bars represent the emission intensities of **HL_1_** in the presence of nitrates of interest (10.0 equiv.). The red bars represent the change in emission upon the subsequent addition of Zn(NO_3_)_2_ (2.0 equiv.) to the above solution; (**b**) emission spectra of **HL_1_** in acetonitrile solution in the presence of an increasing amount of Zn^2+^ in filtered Milli-Q water. Inset: Fluorescence intensity of **HL_1_** depending on the Zn^2+^ in the range from 0 to 2.0 equiv.

The competitive experiments between the **HL_1_**–**HL_6_** probes and coexisting metal ions were investigated on the basis of emission spectra. As shown in Figure 3a, whether in the absence or presence of competitive metal ions, the strong increase in the emission intensity of the **HL_1_** probe was observed upon the addition of Zn^2+^. Although Cd^2+^ and Pb^2+^, among the verified cations, triggered enhancements in emission, these were negligible compared to that of Zn^2+^. When **HL_2_**–**HL_6_** were treated with 10 equiv. of other metal ions and 2 equiv. of Zn^2+^, similar luminescence changes were also observed. The effect was almost the same as that resulting from the addition of Zn^2+^ (Figure S3). In all, the coexistence of these cations had a negligible effect on the detection of Zn^2+^ in the tested media.

#### 2.1.3. Study of the Reversibility and Dependence of Fluorescence on Solvent, pH, Different Zinc Salts

**Figure 4:**
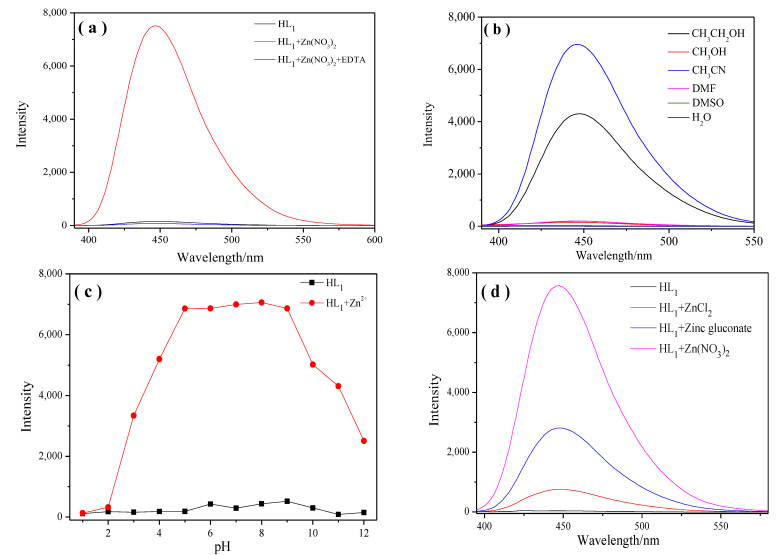
(**a**) Zn^2+^ and EDTA were added to a solution of **HL_1_** in acetonitrile; (**b**) emission spectra of the **HL_1_** probe in different solvents, excitation at 380 nm; (**c**) fluorescence spectra of **HL_1_** interacting with Zn^2+^ at different pH in acetonitrile; (**d**) effects on fluorescence spectra of **HL_1_** using different zinc salts in acetonitrile.

To examine whether the complexation of **HL** and Zn^2+^ is reversible, we added an aqueous solution of Na_2_EDTA during detection. Upon the addition of Na_2_EDTA (1.0 equiv.) to the solution of **HL_1_** containing Zn^2+^, the emission intensity of the mixture solution decreased significantly with the luminescence quenching.t It seems logical that the fluorescent intensity observed in Figure 4a is inversely proportional to the stability of the complex formed by **L**-**Zn** with the anions as the fluorescence of the complex can be quenched by Na_2_EDTA [29]. Changes in the solvent and pH had non-negligible effects on the detection effect of the fluorescent probe. Therefore, we investigated the dependence of the fluorescence emission intensity on solvent and pH during the recognition of Zn^2+^ by **HL_1_**. Interestingly, the intensity of the fluorescence emission spectrum in the acetonitrile solvent was significantly enhanced, and CH_3_CH_2_OH was also a good choice under some conditions (Figure 4b). Further investigation of the effect of pH on the detection of Zn^2+^ was carried out by adjusting the required pH using 0.01 M HCl or 0.01 M NaOH. The fluorescence emission intensity was stable within the pH range of 5.5–9.0, which includes physiological range basically (Figure 4c). Under strong acid or base conditions, the stability of the C=N bond and the presence of -OH can have a significant impact, resulting in fluorescence quenching. To judge whether the probe can selectively recognize Zn^2+^ in different zinc salts, an examination of the sensitivity of the **L_1_** probe to several different zinc salts (zinc chloride, zinc gluconate, and zinc nitrate) was undertaken using fluorescence spectroscopy. However, the fluorescence spectrum enhancement of the **L_1_** probe was not obvious when it was complexed with zinc chloride and zinc gluconate, and the fluorescence enhancement phenomenon only appeared when it interacted with zinc nitrate (Figure 4d). Therefore, we preliminarily speculate that the **HL** probe recognizes Zn^2+^ in the presence of NO_3_^−^, which is involved in the coordination process.

#### 2.1.4. Zn^2+^ Titration Analysis

Fluorescence titration spectra were examined to obtain additional information regarding the binding form of the **HL_1_** probe with Zn^2+^ (Figure 3b). After adding Zn^2+^ to the solution, **HL_1_** exhibited a broad emission profile peaking at 447 nm in acetonitrile solution and displayed a linear enhancement in the emission intensity. The increase in the intensity of the emission spectra stopped after the addition of 1 equivalent of metal ions, which suggested 1:1 binding stoichiometry between **HL_1_** and Zn^2+^ (Figure 3b, inset). In addition, the photoluminescence titration experiments of individual complexes of **HL_2_**–**HL_6_** with Zn^2+^ in varying concentrations were carried out by monitoring the emission intensity changes. Similarly, the emission intensity of **HL_2_**–**HL_6_** increased continuously until the addition of 1 equiv. of Zn^2+^; the further addition of Zn^2+^ induced only minor changes in the luminescence spectra (Figure S4).

### 2.2. Binding Mechanism

According to literature reports [30,31,32], the supramolecular chemical data analysis method was adopted to analyze the combination of host and guest, and the binding constants of host and guest 1:1, 1:2, and 2:1 were simulated, respectively. The results are shown in Table 1. The detection limit of **HL_1_** for Zn^2+^ was calculated as 3*σ*/*k*, where *σ* is the standard deviation of the blank measurement and *k* is the slope of the plot of the emission intensity ratio versus Zn^2+^ concentration [33]. The limit of detection (LOD) of **HL_2_**–**HL_6_** for Zn^2+^ are 2.8 × 10^−8^ M, 2.2 × 10^−6^ M, 3.3 × 10^−8^ M, 1.4 × 10^−7^ M, 2.3 × 10^−8^ M, and 1.74 × 10^−7^ M, respectively. The fluorescence intensity of the **HL** probe had a good linear relationship with the concentration of Zn(NO_3_)_2_ and had a lower detection limit, which could be used for the quantitative detection of Zn(NO_3_)_2_.

**Figure 5:**
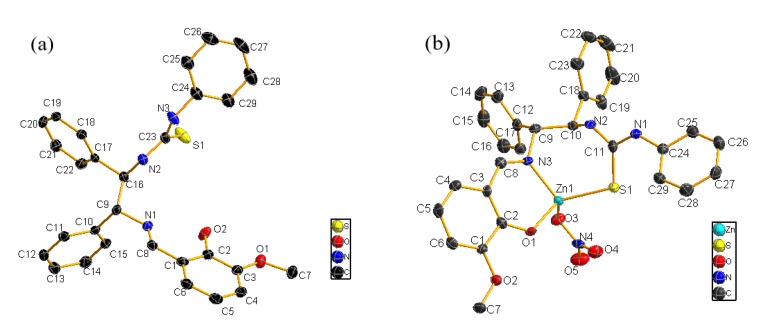
Oka Ridge Thermal Ellipsoidal plot (ORTEP) diagrams of **HL_4_** (**a**) and **L_4_**-ZnNO_3_ (**b**) with the atom numbering schemes at the 40% probability level. Hydrogen atoms and solvent molecules are omitted for clarity.

To further confirm the coordination mechanism of ligand **HL** with zinc nitrate, the crystals were obtained and analyzed. Single crystals of **HL_4_** (CCDC: 2077389) and **L_4_**-ZnNO_3_ (CCDC: 2077390) suitable for X-ray diffraction study were obtained by the slow evaporation of mixed solutions of acetonitrile/methanol at room temperature. **L_4_**-ZnNO_3_ was cultured in proportions of equal amounts and an excessive amount of Zn(NO_3_)_2_, and the complex single crystal with a coordination mode of 1:1 was obtained. The ellipsoid diagrams of **HL_4_** and **L_4_**-ZnNO_3_ shown in Figure 5 were obtained by X-ray single crystal diffraction analysis. The corresponding crystallographic data are summarized in Table S1, and selected bond lengths and angles are listed in Table S2. The single crystal of the complex **L_4_**-ZnNO_3_ clearly showed the formation of a six-membered ring and a seven-membered ring. The central Zn atom adopted a four-coordination method, which was, respectively, connected to the S atom (Zn-S = 2.314(16) Å), the N atom of C=N (Zn-N3 = 2.020(5) Å), the O atom of the salicylaldehyde phenolic hydroxyl group (Zn-O1 = 1.946(5) Å), and the O atom of NO_3_ (Zn-O3 = 2.043(5) Å). This coordination mode is consistent with the coordination mode of monodentate ligands reported in the literature [34,35,36]. The crystal structure indicates that the N atom on the thiourea group was not coordinated, and that only the S atom is involved.

Coordination mechanism studies confirm that **HL** and Zn(NO_3_)_2_ form strongly fluorescent complexes, which suggests 1:1 binding stoichiometry between **HL** and Zn(NO_3_)_2_. Through the single-crystal structure of the complex, it can be visually seen that the atoms coordinated with Zn^2+^ include O atoms provided by phenolic hydroxyl groups, N atoms provided by Schiff bases, and S atoms provided by thiourea structural units. Zn^2+^ with **HL** forms a six-membered ring and a seven-membered ring with coordination atoms.

### 2.3. Theoretical Study

**Figure 6:**
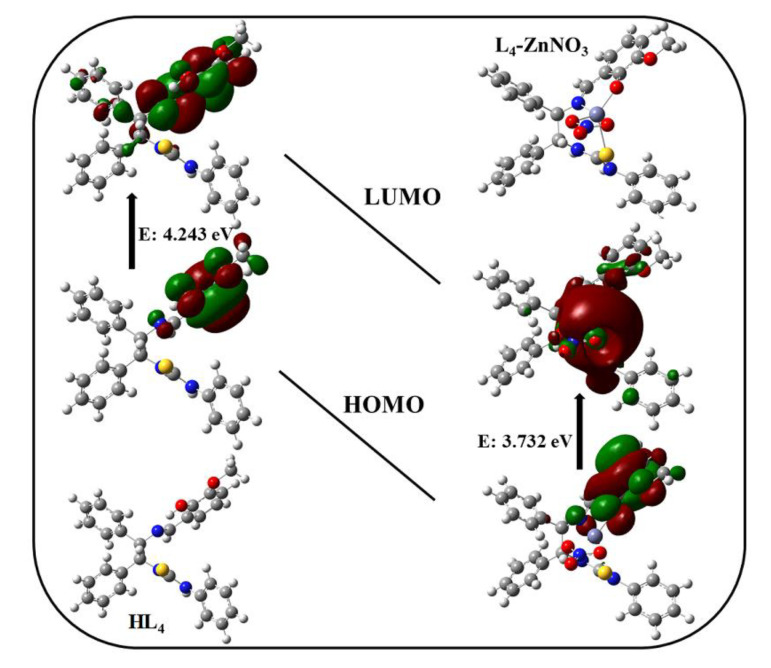
Frontier molecular orbitals of probe **HL_4_** and **L_4_**-ZnNO_3_ as obtained from DFT calculations.

Density Functional Theory (DFT) calculations and time-dependent DFT (TD-DFT) using B3LYP were carried out with Gaussian 09 software package ADDIN EN.CITE [37]. The 6-31G(d,p) basis set was employed for C, H, N, O, and S atoms, and the LANL2DZ basis set was employed for the Zn atom [38,39]. Full geometry optimizations of the **HL_4_** and **L_4_**-**ZnNO_3_** in the singlet ground-state were carried out using the DFT method (Figure 6). The assignment of the type of each MO is made on the basis of its composition. The frontier molecular orbital results from the extension geometries show that the spatial distributions of the HOMO (HOMO = highest occupied molecular orbital) and LUMO (LUMO = lowest unoccupied molecular orbital) are both localized with the phenol moiety with a bandgap of 4.243 eV. However, after the complexation of **HL_4_** with Zn^2+^, the HOMOs of **L_4_**-**ZnNO_3_** were spread over the phenol moiety, and the LUMOs extended to the Zn^2+^ and thiourea moiety with the bandgap of 3.732 eV, which indicates the possible electron transfer from ligand to metal (LMCT). The theoretical bond length between S and O was 5.12262 Å before adding Zn^2+^, and was shortened to 4.05971 Å after forming a complex with **HL_4_**, which was consistent with the change in the crystal data.

### 2.4. Fluorescence Studies of L-ZnNO_3_ in the Presence of Anions

#### 2.4.1. Anion Selectivity Experiments

**Figure 7:**
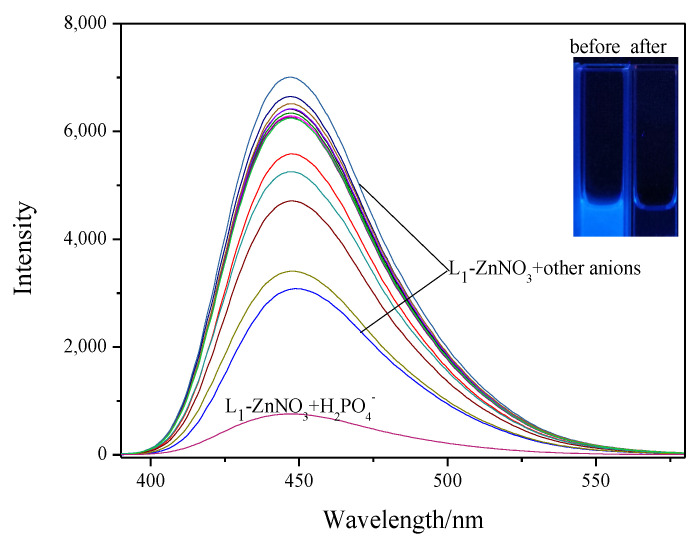
Fluorescence spectra of **L_1_**-Zn^2+^-NO_3_^−^ in acetonitrile with different anions dissolved in filtered Milli-Q water.

Different zinc salt selectivity experiments revealed that different kinds of anions could affect the Zn^2+^ recognition performance. To investigate the effect of different kinds of anions on zinc complexes, anion selectivity experiments were performed. When excited at 377 nm, **L_1_**-ZnNO_3_ displayed a strong emission peak at 449 nm. Among a set of different anions (Cl^−^, Br^−^, F^−^, CH_3_COO^−^, NO_3_^−^, C_6_H_5_COO^−^, C_6_H_5_SO_2_^−^, PO_4_^2−^, H_2_PO_4_^−^, HPO_4_^2−^, SO_3_^2−^, SO_4_^2−^, H_2_PO_2_ ^−^, NO_2_^−^, NO_3_^−^, and SCN^−^), a prominent change in the emission spectra of **L_1_**-ZnNO_3_ manifested upon the addition of H_2_PO_4_^−^ alone (Figure 7). When **L_1_**-ZnNO_3_ was combined with H_2_PO_4_^−^, the bright blue color of the solution disappeared, resulting in a colorless transparent solution (Figure 7, insert and Figure S5). A similar phenomenon also occurred when adding H_2_PO_4_^−^ to **L_2_**-ZnNO_3,_ **L_3_**-ZnNO_3_, **L_4_**-ZnNO_3_, **L_5_**-ZnNO_3_, and **L_6_**-ZnNO_3_ (Figure S6). The above experimental results proved that this type of fluorescent probe could realize the “turn-on–turn-off” behavior, thus realizing the recognition function of cation and anion.

#### 2.4.2. Anion-Competitive Experiments

**Figure 8:**
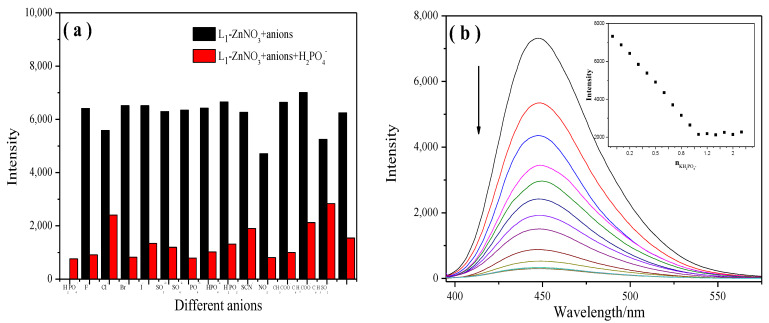
(**a**) Comparison of fluorescence intensity before and after the addition of **L_1_**-ZnNO_3_ in acetonitrile; (**b**) fluorescence emission spectra of **L_1_**-ZnNO_3_ in acetonitrile solution with the addition of H_2_PO_4_^−^. Inset: Fluorescence intensity of **L_1_**-ZnNO_3_ depending on the H_2_PO_4_^−^ in the range from 0 to 2.0 equiv.

To verify the influence of other anions on the probe recognition of H_2_PO_4_^−^, the emission intensity of the probe and H_2_PO_4_^−^ at 450 nm (λ_ex_ = 370 nm) was monitored (Figure 8a). The result showed that the **L_1_**-ZnNO_3_ could specifically detect H_2_PO_4_^−^ in the presence of other related anions. The anion-competitive experiments of **L_2_**-ZnNO_3,_ **L_3_**-ZnNO_3_, **L_4_**-ZnNO_3_, **L_5_**-ZnNO_3_, and **L_6_**-ZnNO_3_ are shown in the Supplementary Materials (Figure S7), which were generally consistent with **L_1_**-ZnNO_3_.

The titration of **L_1_**-ZnNO_3_ with H_2_PO_4_^−^ triggered a distinct decrease in the emission intensity at 450 nm (Figure 8b). **L_2_**-ZnNO_3_~**L_6_**-ZnNO_3_ and H_2_PO_4_^−^ are exhibited in the Supplementary Materials (Figure S8).

In this work, a series of novel chiral thiourea fluorescent probes **HL_1_**–**HL_6_** were prepared for the first time for fluorescence-responsive Zn^2+^, while their complex **L-Zn-NO_3_** was continuously identified and detected the phosphate anion (H_2_PO_4_^−^). Compared with other working probes for the detection of analytes (Table S4) [7,40,41,42,43,44,45,46], the probes synthesized in this work have the advantages of high sensitivity and low detection limits.

## 3. Materials and Methods

### 3.1. General Procedures

#### 3.1.1. Reagents and Instruments

All of the materials used for synthesis were purchased from commercial suppliers and used as received. Nitrate salts of Ag^+^, Na^+^, K^+^, Co^2+^, Fe^3+^, Cu^2+^, Cd^2+^, Cr^3+^, Mg^2+^, Pb^2+^, Ni^2+^, and Zn^2+^, sulfate salts of Fe^2+^ and Hg^2+^, and chlorine salt of Ca^2+^ were used for the spectroscopic studies. The different sodium salts of F^−^, Cl^−^, Br^−^, CH_3_COO^−^, C_6_H_5_COO^−^, C_6_H_5_SO_2_^−^, PO_4_^3−^, H_2_PO_4_^−^, HPO_4_^2−^, SO_3_^2−^, SO_4_^2−^, H_2_PO_2_^−^, NO_2_^−^, NO_3_^−^, and SCN^−^ were used for investigating anion effects. All salts were dissolved in filtered Milli-Q water to prepare aqueous ion solutions.

The melting points of the products were determined on an X-4 binocular microscope. ^1^H and ^13^C NMR spectra were recorded on a Bruker 400 MHz instrument at room temperature. Chemical shifts were measured relative to residual solvent peaks of CDCl_3_ (^1^H: δ = 7.26 ppm; ^13^C: δ = 77.0 ppm) or DMSO-*d*_6_ (^1^H: δ = 2.50 ppm; ^13^C: δ = 39.5 ppm) with tetramethylsilane (TMS) as internal standard. The following abbreviations are used to describe spin multiplicities in ^1^H NMR spectra: s = singlet; d = doublet; t = triplet; m = multiplet. Specific rotations were measured on a PerkinElmer 341 MC polarimeter. HRMS data were obtained using a 7.0T FT-ICR MS spectrometer. X-ray crystal diffraction measurements of **L** were acquired using a Bruker SMART APEX II CCD diffractometer. Fluorescence spectra were examined on a Hitachi FL-2700 spectrophotometer using 10 mm path length quartz cuvettes at 298 K. The pH was calculated by STARTER 3100/F.

#### 3.1.2. Synthesis of **HL_1_**–**HL_6_** and Compound **3**

The synthetic route for **HL_1_**–**HL_6_** and compound **3** is shown in Figure 1. Under ice-cold conditions, (1*R*,2*R*)-1,2-diphenylethylenediamine (**1**, 4.71 mmol) was dissolved in dichloromethane under ice-cold conditions with constant stirring. Isothiocyanate (4.71 mmol) was dissolved in dichloromethane and then added dropwise to the above amine system via a constant pressure dropping funnel. The reaction mixture was stirred for 12 h and then evaporated under low pressure. The crude product was purified by silica gel column chromatography using dichloromethane as an eluent to give the chiral primary amine thiourea **2** in the form of a white powder. Compound **2** and the substituted aldehydes were dissolved in an ethanol solvent and heated to reflux under nitrogen protection for 24 h. The mixture was cooled and washed with ether (3 × 50 mL) to give the solid products **HL_1_**–**HL_6_** and compound **3**.

1-((1*R*,2*R*)-2-(((*E*)-2-Hydroxybenzylidene)amino)-1,2-diphenylethyl)-3-phenylthiourea (**HL_1_**). White solid, 70% yield, m.p. 104–108 °C; ${\left[\alpha \right]}_{D}^{23}$ = +33.44 (*c* = 10, CH_2_Cl_2_); ^1^H NMR (CDCl_3_, 400 MHz) δ 12.54–12.23 (m, 1H), 8.06 (s, 1H), 7.69 (s, 1H), 7.48–7.28 (m, 7H), 7.24–6.74 (m, 13H), 5.99 (s, 1H), 4.81 (d, *J* = 4.1 Hz, 1H); ^13^C NMR (CDCl_3_, 101 MHz) δ 180.5, 167.2, 160.6, 138.6, 138.2, 137.4, 135.7, 133.0, 132.0, 130.3, 129.3, 128.5, 128.3, 127.9, 127.6, 127.3, 127.2, 126.3, 125.4, 124.0, 118.9, 118.4, 117.0, 64.2, 37.1, 34.4, 32.7, 31.9, 30.1, 29.7, 29.4, 26.9, 22.7, 14.1, 11.4; HRMS (ESI) *m*/*z* calc’d for C_28_H_25_N_3_OS [M + H]^+^: 452.1797, found 452.1790.

1-((1*R*,2*R*)-2-(((*E*)-2,3-Dihydroxybenzylidene)amino)-1,2-diphenylethyl-3-phenylthiourea (**HL_2_**). Yellow solid, 48% yield, m.p. 146–149 °C; ${\left[\alpha \right]}_{D}^{23}$ = +36.61 (*c* = 10, CH_2_Cl_2_); ^1^H NMR (DMSO-*d*_6__,_ 400 MHz) δ 13.20 (d, *J* = 155.7 Hz, 1H), 9.88 (s, 1H), 9.12 (s, 1H), 8.75–8.36 (m, 2H), 7.59–6.54 (m, 18H), 6.11 (t, *J* = 8.3 Hz, 1H), 5.15–4.87 (m, 1H); ^13^C NMR (DMSO-*d*_6__,_ 101 MHz) δ 180.4, 166.9, 149.1, 145.5, 140.2, 139.8, 139.4, 128.7, 128.4 (d, *J* = 10.9 Hz), 128.3, 127.8, 127.6, 127.3, 126.9, 126.7, 123.9, 122.6, 121.9, 118.8, 118.4, 118.3, 76.5, 62.8; HRMS (ESI) *m*/*z* calc’d for C_28_H_25_N_3_O_2_S [M + H]^+^: 468.1746, found 468.1749.

1-((1*R*,2*R*)-2-(((*E*)-2-Hydroxy-5-methylbenzylidene)amino)-1,2-diphenylethyl)-3-phe-nylthiourea (**HL_3_**). White solid, 81% yield, m.p. 139–142 °C; ${\left[\alpha \right]}_{D}^{23}$ = +42.78 (c = 10, CH_2_Cl_2_); ^1^H NMR (DMSO-*d*_6_, 400 MHz) δ 12.45 (s, 1H), 9.75 (s, 1H), 8.48–8.16 (m, 2H), 7.39–7.03 (m, 17H), 6.82 (d, *J* = 8.3 Hz, 1H), 6.14 (t, *J* = 7.6 Hz, 1H), 4.93 (d, *J* = 6.5 Hz, 1H), 2.22 (s, 3H); ^13^C NMR (DMSO-*d*_6_, 101 MHz) δ 178.1, 164.0, 155.3, 138.1, 137.7, 136.8, 131.1, 129.1, 126.2, 126.0, 125.6, 125.3, 125.2, 125.2, 125.0, 124.6, 121.9, 120.6, 116.2, 114.0, 74.3, 60.5, 17.6; HRMS (ESI) *m*/*z* calc’d for C_29_H_27_N_3_OS [M + H]^+^: 466.1953, found 466.1958.

1-((1*R*,2*R*)-2-(((*E*)-2-Hydroxy-3-methoxybenzylidene)amino)-1,2-diphenylethyl)-3-p-henylthiourea (**HL_4_**). Brown solid, 84% yield, m.p. 150–152 °C; ${\left[\alpha \right]}_{D}^{23}$ = +48.81 (*c* = 10, CH_2_Cl_2_); ^1^H NMR (DMSO-*d*_6_, 400 MHz) δ 12.55 (s, 1H), 9.35 (s, 1H), 8.27–7.34 (m, 2H), 7.23–6.53 (m, 17H), 6.44 (d, *J* = 7.9 Hz, 1H), 5.74 (s, 1H), 4.55 (d, *J* = 6.6 Hz, 1H), 3.39 (s, 3H); ^13^C NMR (DMSO-*d*_6_, 101 MHz) δ 180.4, 166.6, 150.1, 147.8, 140.2, 139.9, 139.1, 128.5, 128.3, 127.9, 127.6, 127.5, 127.4, 126.9, 124.1, 123.0, 122.8, 118.6, 118.4, 115.0, 76.3, 62.7, 55.8; HRMS (ESI) *m*/*z* calc’d for C_29_H_27_N_3_O_2_S [M + H]^+^: 482.1902, found 482.1907.

1-((1*R*,2*R*)-2-((*(E*)-5-Chloro-2-hydroxybenzylidene)amino)-1,2-diphenylethyl)-3-phe-nylthiourea (**HL_5_**). Yellow solid, 80% yield, m.p. 158–160 °C; ${\left[\alpha \right]}_{D}^{23}$ = +37.11 (*c* = 10, CH_2_Cl_2_); ^1^H NMR (DMSO-*d*_6_, 400 MHz) δ 12.58 (s, 1H), 9.73 (s, 1H), 8.30 (d, *J* = 8.0 Hz, 2H), 7.54 (s, 1H), 7.40–7.14 (m, 16H), 7.09 (d, *J* = 7.2 Hz, 1H), 6.94 (d, *J* = 8.8 Hz, 1H), 6.14 (s, 1H), 4.93 (d, *J* = 6.3 Hz, 1H); ^13^C NMR (DMSO-*d*_6_, 101 MHz) δ 180.5, 164.3, 158.4, 140.2, 132.3, 129.9, 128.5, 128.3, 127.9, 127.6, 127.5, 127.4, 127.0, 123.0, 122.4, 120.3, 118.5, 76.5, 62.7; HRMS (ESI) *m*/*z* calc’d for C_28_H_24_ClN_3_OS [M + H]^+^: 482.1407, found 486.1412.

1-((1*R*,2*R*)-2-(((*E*)-(2-Hydroxynaphthalen-1-yl)methylene)amino)-1,2-diphenylethyl)-3-phenylthiourea (**HL_6_**). Yellow solid, 74% yield, m.p. 154–156 °C; ${\left[\alpha \right]}_{D}^{23}$ = +46.22 (*c* = 10, CH_2_Cl_2_); ^1^H NMR (DMSO-*d*_6_, 400 MHz) δ 14.92 (s, 1H), 9.65 (s, 1H), 9.25 (s, 1H), 8.42 (s, 1H), 8.04 (s, 1H), 7.93–7.71 (m, 2H), 7.49 (s, 1H), 7.36–7.06 (m, 15H), 6.96 (d, *J* = 9.2 Hz, 2H), 6.22 (s, 1H), 5.24 (s, 1H); ^13^C NMR (DMSO-*d*_6_, 101 MHz) δ 180. 4, 161.3, 139.3, 138.9, 135.9, 133.1, 129.0, 128. 5, 128.0, 127. 9, 127.8, 127.6, 127.5, 127.2, 126.3, 122.9, 123.0, 122.2, 119.2, 107.2, 72.7; HRMS (ESI) *m*/*z* calc’d for C_32_H_27_N_3_OS [M + H]^+^: 502.1953, found 502.1959.

1-((1*R*,2*R*)-2-(((*E*)-Benzylidene)amino)-1,2-diphenylethyl)-3-phenylthiourea (**3**). White solid, 76% yield, m.p. 175–179 °C; ${\left[\alpha \right]}_{D}^{23}$ = +27.33 (*c* = 10, CH_2_Cl_2_); ^1^H NMR (CDCl_3_, 400 MHz) δ 7.99–7.76 (m, 2H), 7.73–7.27 (m, 16H), 7.26–7.02 (m, 5H), 5.92 (s, 1H), 4.60 (s, 1H); ^13^C NMR (CDCl_3_, 101 MHz) δ 180.4, 162.9, 141.2, 140.5 (d, *J* = 65.6 Hz), 135.3, 131.2, 130.2, 128.5, 128.4, 128.3, 127.6, 127.4, 127.2, 126.7, 126.1, 77.3, 64.9; HRMS (ESI) *m*/*z* calc’d for C_28_H_25_N_3_S [M + H]^+^: 436.1847, found 436.1841.

### 3.2. Spectroscopic Measurements

Stock solutions of various ions (0.50 mol/L) were prepared in filtered Milli-Q water. A stock solution of **HL** probe (1.0 × 10^−2^ mol/L) and compound **3** in acetonitrile was freshly prepared for fluorescence measurement. Fluorescence spectroscopic measurements were recorded for samples at 1 min after the addition of various analytes. All spectroscopic measurements were performed at 25 °C. For fluorescence measurements, the excitation wavelength was fixed at 380 nm (λ_ex_ = 380 nm) and 357 nm for **HL_1_** and **HL_2_**, and at 390 nm (λ_ex_ = 390 nm) for **HL_3_**–**HL_6_** and compound **3**, respectively. The emission wavelength was recorded from 220 to 600 nm for **HL_1_**–**HL_6_** and compound **3**. In the cation selectivity experiments, the test samples were prepared by interacting 20 μL of the cation stock (0.50 mol/L) with 1 mL of **HL** solution (1.0 × 10^−4^ mol/L) and compound **3**. In the presence of other metal ions, the competition between Zn^2+^ and other metal ions was systematically studied by fluorescence emission spectroscopy. In the titration experiments, the solution of **HL** (1.0 × 10^−5^ mol/L) was placed in a quartz cuvette, and a certain volume of the metal ion stock solution (5.0 × 10^−4^ mol/L) was added gradually to achieve a concentration of 1.0 × 10^−5^ mol/L. Similarly, anion experiments were conducted based on **L**-ZnNO_3_ to study the fluorescence spectra after combination with different anions.

### 3.3. Synthesis of the Sensor Zn(II) Complex and X-ray Crystallography

A methanol solution (2 mL) of Zn(NO_3_)_2_·6H_2_O (0.0297 g, 1 equiv.) was added into the round bottom flask containing acetonitrile (3 mL) and compound **HL_4_** (0.0482 g, 0.1 mmol). Then, it was sealed with unsintered polytetrafluoroethylene (PTFE) tapes and holes were pricked in the tape with a needle. The crystals of **L_4_**-ZnNO_3_ were obtained after the solvent slowly evaporated at room temperature.

The crystal structures were determined using a Bruker SMART APEX II CCD diffractometer with a monochromator and Cu Kα radiation (λ = 0.71073 Å) at 114(2) or 146.4(3) K with an increasing ω (width of 0.3° per frame) at a scan speed of 5 s per frame. Using Olex2 [47], the structure was solved with the ShelXT [48] structure solution program using Direct Methods and refined with the ShelXL [49] refinement package using least squares minimization. All non-hydrogen atoms were refined anisotropically and the hydrogen atoms attached to all carbon atoms were geometrically fixed. The positional and temperature factors were refined isotropically.

## 4. Conclusions

Chiral thiourea Schiff base compounds were synthesized and their binding properties with cations were studied. The spectral data showed that the **HL_1_**–**HL_6_** probes have high selectivity and sensitivity to Zn^2+^ and are not affected by the presence of other coexisting metal ions. Combined with the crystal data, it was determined that the **HL** and Zn(NO_3_)_2_ form a **L**-ZnNO_3_ complex in a 1:1 ratio. The formed **L**-ZnNO_3_ complex can selectively recognize H_2_PO_4_^−^ without interference from other anions. The probe has good prospects for application and provides a new approach for the detection of Zn(NO_3_)_2_ and H_2_PO_4_^−^ in actual samples.

## Figures and Tables

**Table 1:** Job plot shapes of **HL_1_** at various concentrations and *K*^1:1^, *K*^1:2^ to *K^2^*^:1^ ratios. *K*^1:1^ = 1000, *K*^1:2^ = 100, *K*^2:1^ = 100 were assumed.

Parameter (Bounds)	Optimized	Error	Initial
*K* (0 → ∞)	8607.30 M^−1^	±9.1006%	100.00 M^−1^
*K*_11_ (0 → ∞)	3376.76 M^−1^	±5.2092%	1000.00 M^−1^
*K*_12_ (0 → ∞)	4790.08 M^−1^	±26.9494%	100.00 M^−1^
*K*_11_ (0 → ∞)	3576.53 M^−1^	±9.1090%	1000.00 M^−1^
*K*_21_ (0 → ∞)	−277,004.73 M^−1^	±−4.6365%	100.00 M^−1^

## Data Availability

All data used to support the findings of this study are included within the article and Supplementary Materials.

## Supplementary Materials

The following supporting information can be downloaded at https://www.mdpi.com/article/10.3390/molecules28104166/s1: Section S1: Fluorescence spectra and photos of **L** with metal ion (Figures S1–S4); Section S2: Fluorescence spectra and photos of **L**-ZnNO_3_ with anion (Figures S5–S8); Section S3: Crystallographic data (Tables S1–S3); Section S4: Examples for detection of Zn^2+^ and phosphate anions by sensors (Table S4); Section S5: ^1^H NMR and ^13^C NMR spectra of **HL_1_**–**HL_6_** and **3** (Figures S9–S22).

## Sample Availability

Samples of the compounds **HL_1_**–**HL_6_** and **3** are available from the authors.
