# Supplementary material for: A Highly Selective and Sensitive Sequential Recognition Probe Zn2+ and H2PO4− Based on Chiral Thiourea Schiff Base
Source: Molecules. 2023 May 18;28(10):4166. doi: 10.3390/molecules28104166 (PMC10221379; doi:10.3390/molecules28104166)
Supplement: Supplementary file 1 [file molecules-28-04166-s001.zip › molecules-2408434-supplementary.pdf]

Supporting Information

A Highly Selective and Sensitive Sequential Recognition Probe Zn^{2+} and $H_2PO_4^-$ Based on Chiral Thiourea Schiff Base

Shan Yang [1,†], Yichuan Huang [1,†], Aidang Lu [1,*], Ziwen Wang [2,*], Hongyan Li [1,*]

[1] School of Chemical Engineering and Technology, Hebei University of Technology, Tianjin 300130, China
[2] Tianjin Key Laboratory of Structure and Performance for Functional Molecules, College of Chemistry, Tianjin Normal University, Tianjin 300387, China

Table of Contents

Section S1 Fluorescence spectra and photos of **HL** with metal ion……… ……. ……. ……. …. …...S4

Figure S1. Emission spectra of probes in acetonitrile solution with different nitrates…S4

Figure S2. Photo of the probes with various nitrates taken at 365 nm……………….…..S5

Figure S3. Fluorescence intensities of probes and Zn^{2+} in the presence of interfering ions………………………………………….………………………………………S6

Figure S4. Fluorescence emission spectra of probes in acetonitrile solution with the addition of Zn^{2+} ……….……………………………… ………………………......S6

Section S2 Fluorescence spectra and photos of **L**-$ZnNO_3$ with anion…………………………………...S7

Figure S5. The color of **L**$_1$-$ZnNO_3$ solution with the addition of $H_2PO_4^-$ under UV lamp………………………………….……………………………………….....S7

Figure S6. Fluorescence spectra of **L**-$ZnNO_3$ with different anion responses………….S7

Figure S7. Changes in fluorescence intensity after adding other anions and $H_2PO_4^-$ of **L**-$ZnNO_3$........S8

Figure S8. Fluorescence emission spectra of complexes in acetonitrile solution with the addition of $H_2PO_4^-$........S8

Section S3 Crystallographic data........S9

Table S1. Crystallographic data for **HL4** probe and **L4**-$ZnNO_3$........S9

Table S2. Selected bond distances (Å) and angles (◦) for **HL4**. S10

Table S3. Selected bond distances (Å) and angles (◦) for complexe **L4**-$ZnNO_3$. S11

Section S4 Examples for detection of Zn^{2+} and phosphate anions by sensors........S12

Table S4. Examples for detection of Zn^{2+} and phosphate anions by sensors... ....S12

Section S5 ^1H NMR and ^{13}C NMR spectra of **HL1–HL6** and **3**........S13

Figure S9. ^1H NMR spectrum of **HL1**........S13

Figure S10. ^{13}C NMR spectrum of **HL1**........S13

Figure S11. ^1H NMR spectrum of **HL2**........S14

Figure S12. ^{13}C NMR spectrum of **HL2**........S14

Figure S13. ^1H NMR spectrum of **HL3**........S15

Figure S14. ^{13}C NMR spectrum of **HL3**........S15

Figure S15. ^1H NMR spectrum of **HL4**........S16

Figure S16. ^{13}C NMR spectrum of **HL4**........S16

Figure S17. ^1H NMR spectrum of **HL5**........S17

Figure S18. ^{13}C NMR spectrum of **HL5**........S17

Figure S19. ^1H NMR spectrum of **HL6**........S18

Figure S20. ^{13}C NMR spectrum of **HL_6**……………………………………………………………S18

Figure S21. ^{1}H NMR spectrum of **3**.............. …………………………………………………………S19

Figure S22. ^{13}C NMR spectrum of **3**……………………………………………………………………S19

Section S1 Fluorescence spectra and photos of **HL** with metal ion

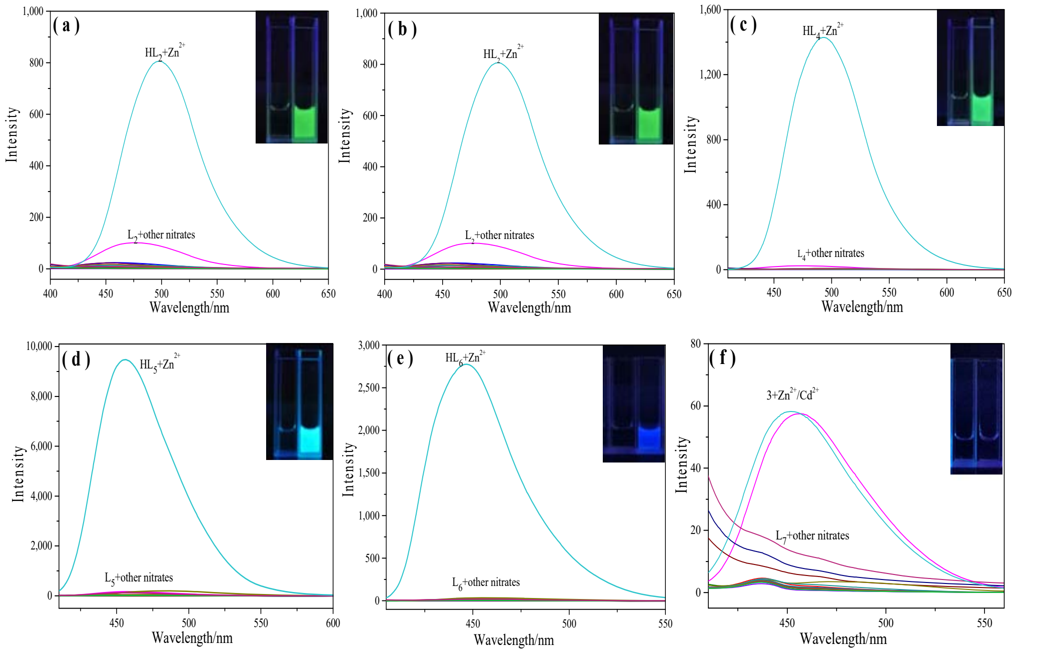

Figure S1. Emission spectra of probes in acetonitrile solution with different nitrates. (a) **HL$_2$**; (b) **HL$_3$**; (c) **HL$_4$**; (d) **HL$_5$**; (e) **HL$_6$**; (f) **3**. Inset: Light change upon the addition of Zn^{2+}.

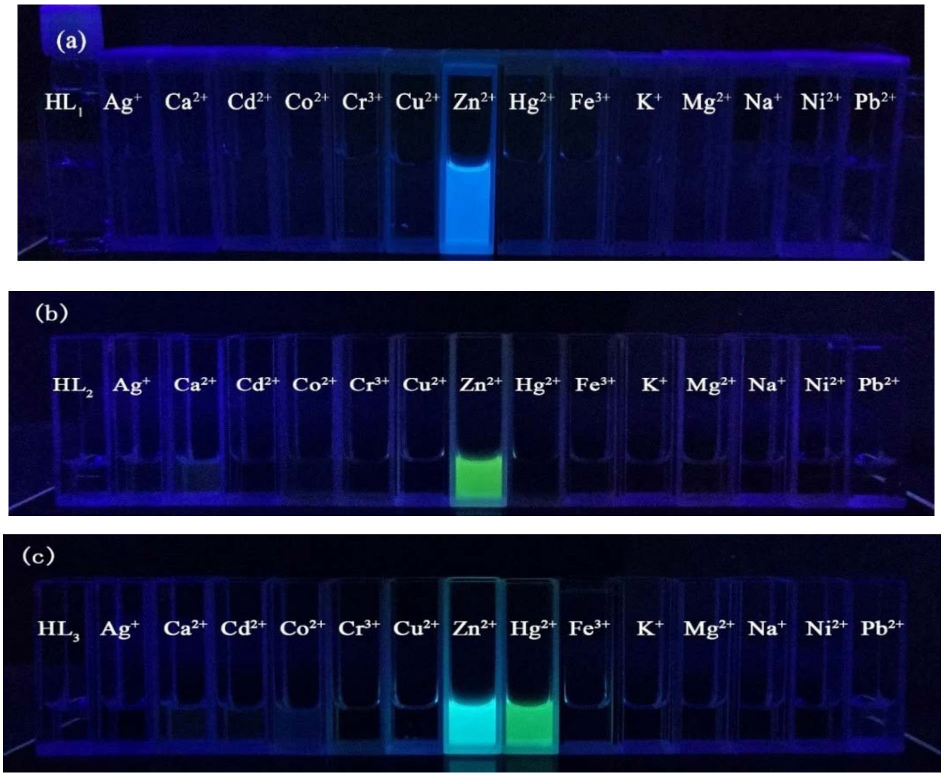

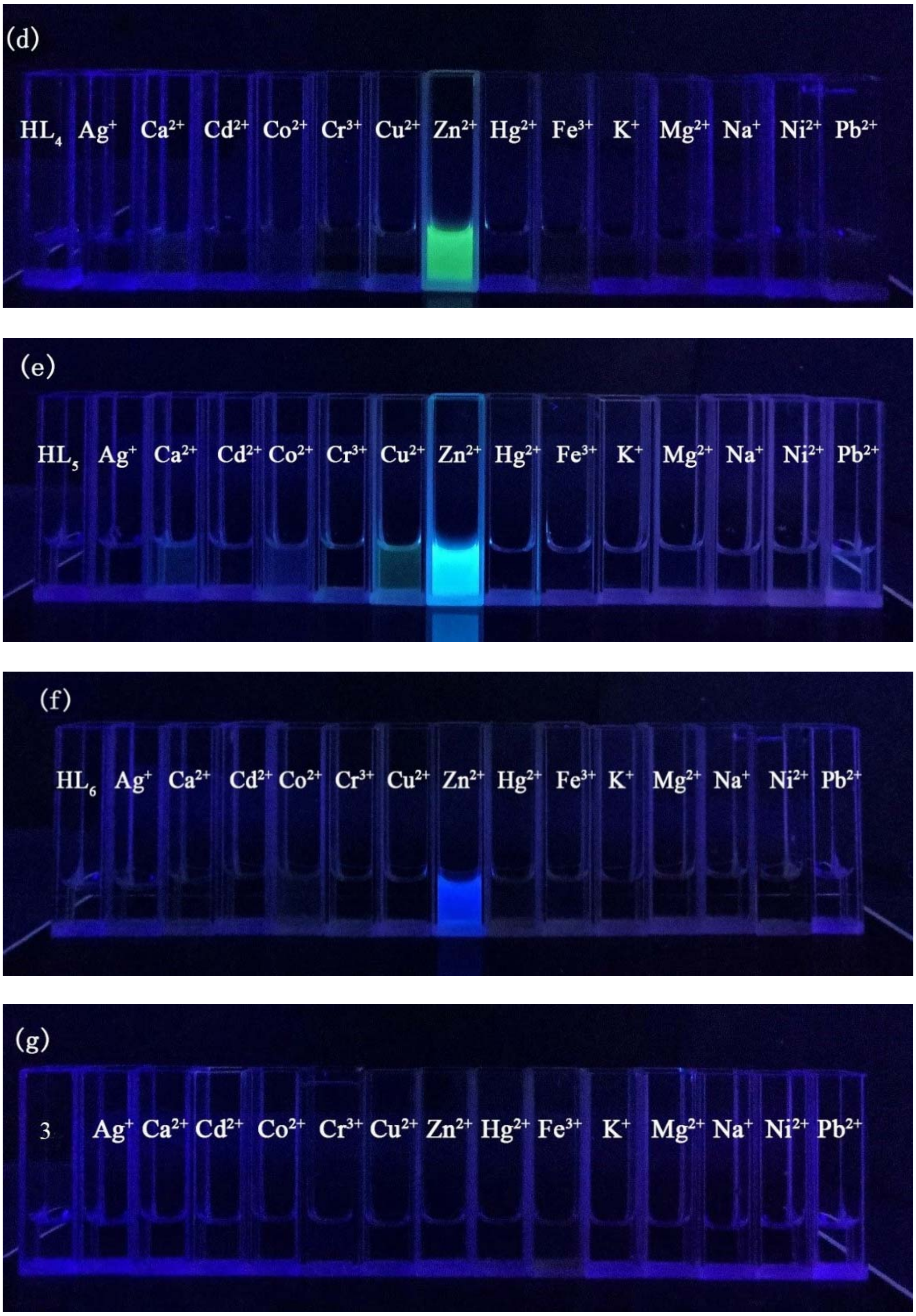

Figure S2. Photo of the probes with various nitrates taken at 365 nm. (a) $\mathbf{HL_1}$; (b) $\mathbf{HL_2}$; (c) $\mathbf{HL_3}$; (d) $\mathbf{HL_4}$; (e) $\mathbf{HL_5}$; (f) $\mathbf{HL_6}$; (g) **3**.

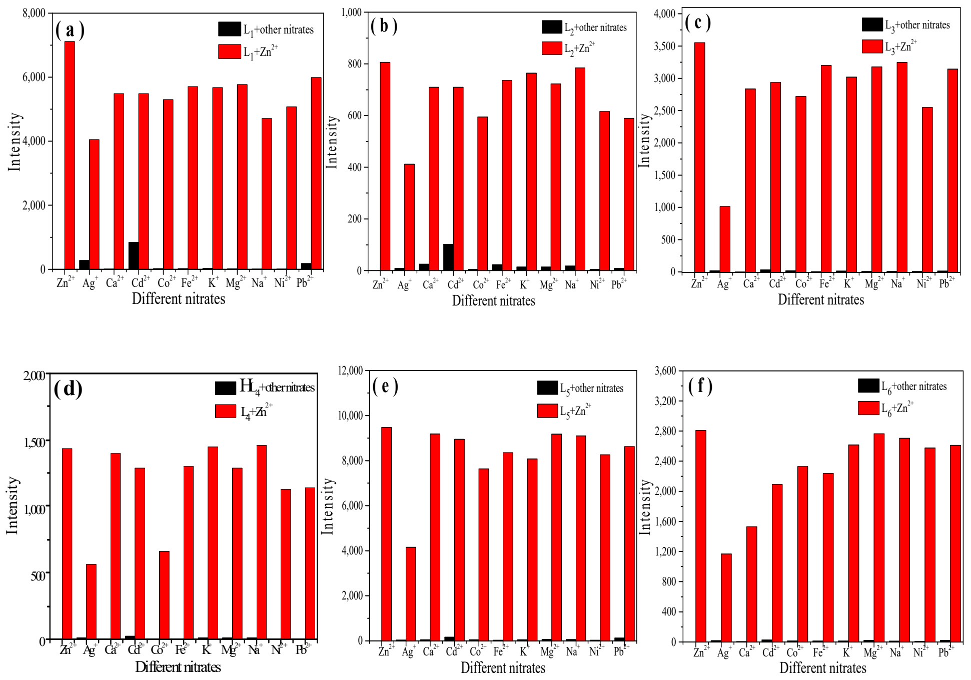

Figure S3. Fluorescence intensities of probes and Zn^{2+} in the presence of interfering ions. (a) **HL_1**; (b) **HL_2**; (c) **HL_3**; (d) **HL_4**; (e) **HL_5**; (f) **HL_6.** The black bars represent the emission intensities of **HL_1-HL_6** in the presence of nitrates of interest. The red bars represent the change of emission upon subsequent addition of Zn^{2+} to the above solution.

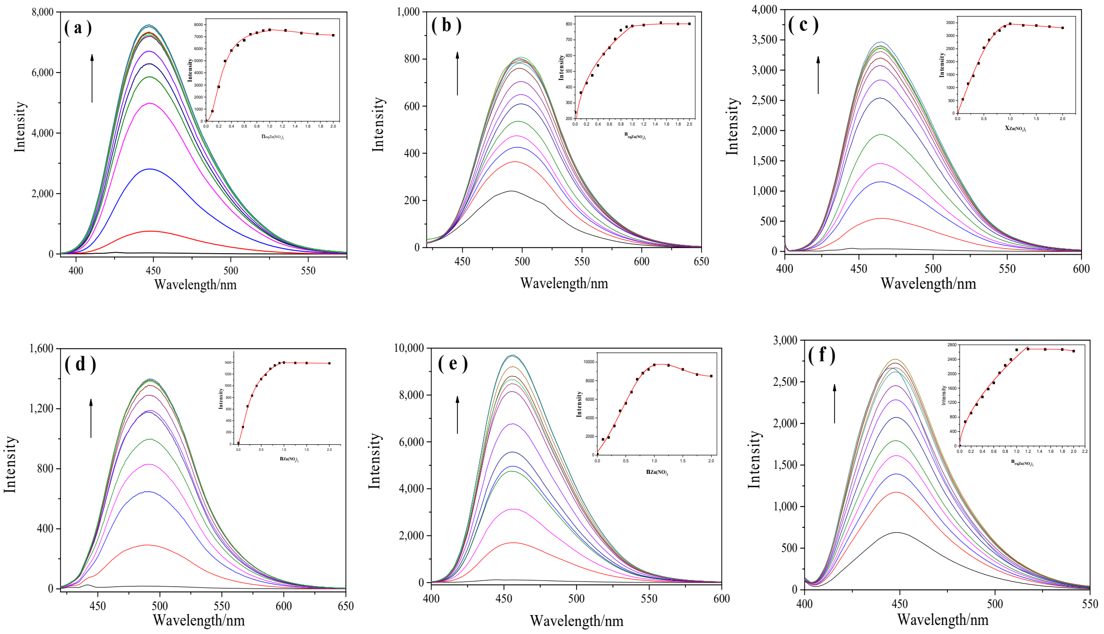

Figure S4. Fluorescence emission spectra of probes in acetonitrile solution with the addition of Zn^{2+}. (a) **HL_1**; (b) **HL_2**; (c) **HL_3**; (d) **HL_4**; (e) **HL_5**; (f) **HL_6**. Inset: Fluorescence intensity of **HL_1-HL_6** depending on the Zn^{2+} in the range from 0 to 2.0 equiv.

Section S2 Fluorescence spectra and photos of **L**-$ZnNO_3$ with anion

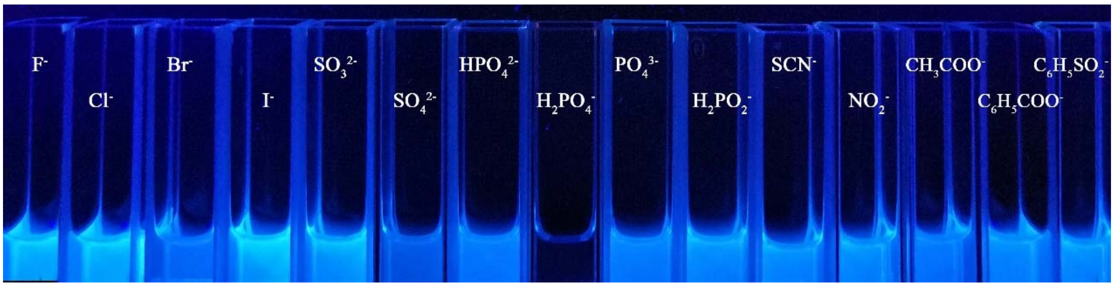

Figure S5. The color of **L1**-$ZnNO_3$ solution with the addition of $H_2PO_4^-$ under UV lamp.

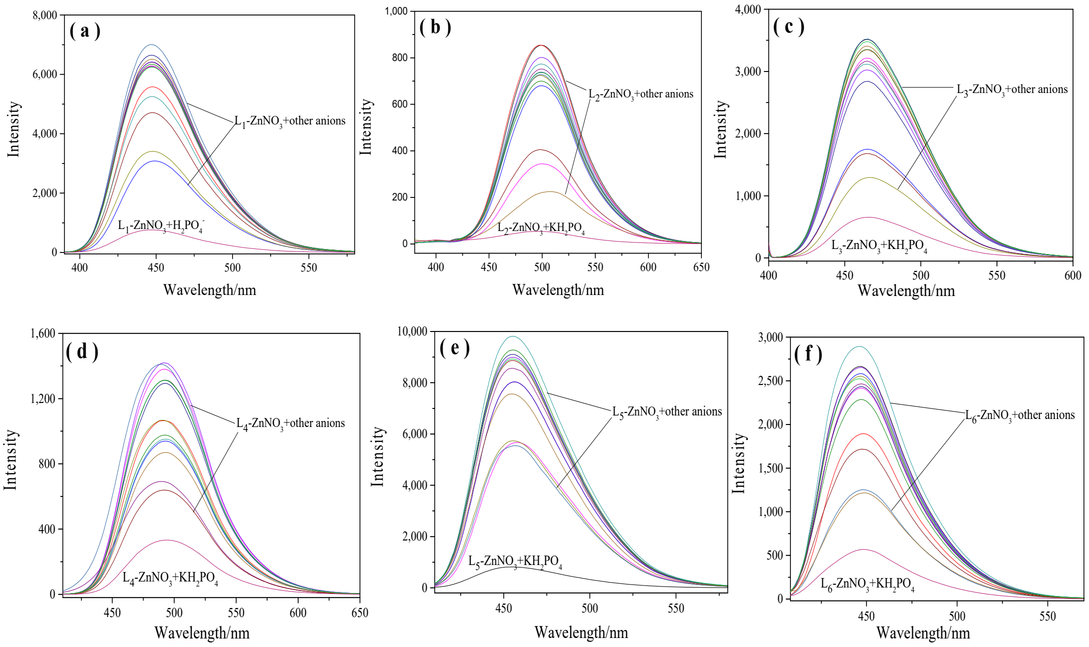

Figure S6. Fluorescence spectra of (a) **L1**-$ZnNO_3$; (b) **L2**-$ZnNO_3$; (c) **L3**-$ZnNO_3$; (d) **L4**-$ZnNO_3$; (e) **L5**-$ZnNO_3$; (f) **L6**-$ZnNO_3$ with different anion responses.

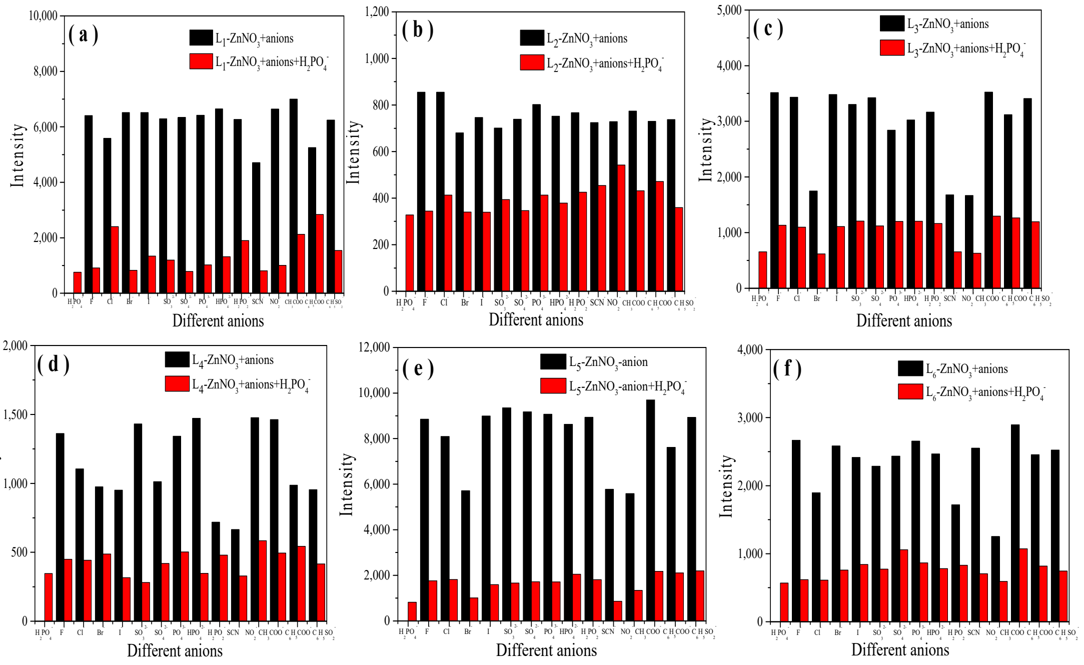

Figure S7. Changes in fluorescence intensity after adding other anions and $H_2PO_4^-$ of (a) $\mathbf{L_1}$-$ZnNO_3$; (b) $\mathbf{L_2}$-$ZnNO_3$; (c) $\mathbf{L_3}$-$ZnNO_3$; (d) $\mathbf{L_4}$-$ZnNO_3$; (e) $\mathbf{L_5}$-$ZnNO_3$; (f) $\mathbf{L_6}$-$ZnNO_3$.

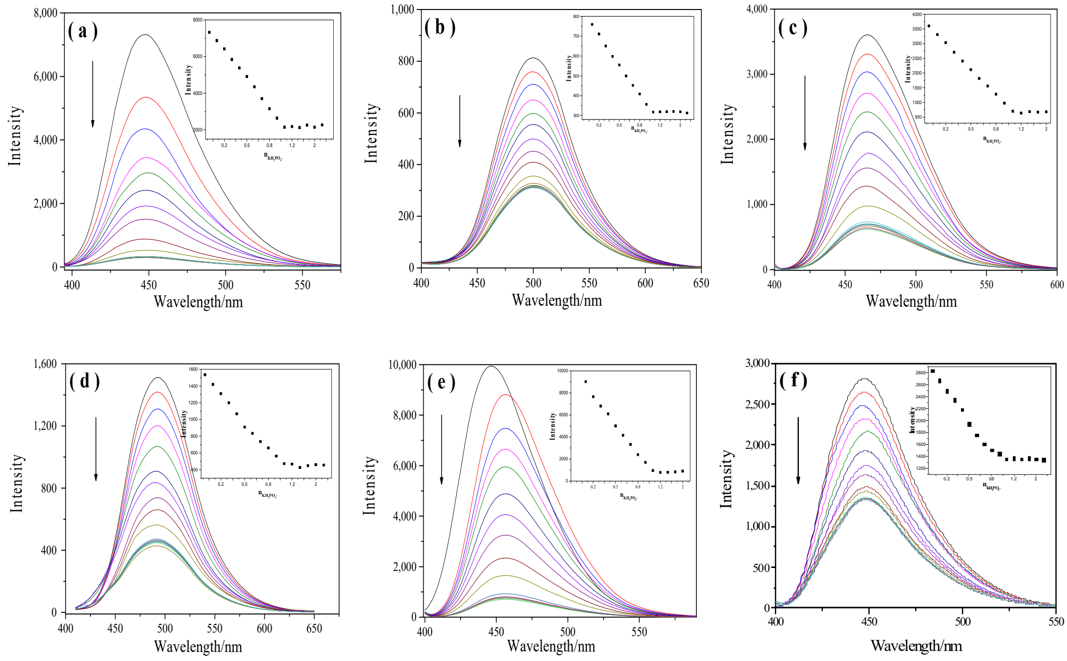

Figure S8. Fluorescence emission spectra of complexes in acetonitrile solution with the addition of $H_2PO_4^-$. (a) $\mathbf{L_1}$-$ZnNO_3$; (b) $\mathbf{L_2}$-$ZnNO_3$; (c) $\mathbf{L_3}$-$ZnNO_3$; (d) $\mathbf{L_4}$-$ZnNO_3$; (e) $\mathbf{L_5}$-$ZnNO_3$; (f) $\mathbf{L_6}$-$ZnNO_3$. Inset: Fluorescence intensity of $\mathbf{L_1}$-$ZnNO_3$~$\mathbf{L_6}$-$ZnNO_3$ depending on the $H_2PO_4^-$ in the range from 0 to 2.0 equiv.

Section S3 Crystallographic data

Table S1. Crystallographic data for the H**L₄** probe and **L₄**-ZnNO₃

	**HL₄**	**L₄**-ZnNO₃
Formula	$C_{29}H_{27}N_3O_2S$	$C_{29}H_{26}N_4O_5SZn$
W	481.59	628.49
T (K)	114(2)	146.4(3)
Crystal syst	Orthorhombic	orthorhombic
Space group	$P2_12_12_1$	$P2_12_12_1$
a (Å)	6.10553(10)	10.8996(3)
b (Å)	19.9507(4)	12.2050(3)
c (Å)	20.2790(4)	42.2958(9)
α (°)	90	90
β (°)	90	90
γ (°)	90	90
V(Å^3)	2470.17(8)	5626.6(3)
Z	4	8
ρ_{calc} (g/cm^3)	1.295	1.484
μ (Mo Kα) (mm^{-1})	1.412	2.314
F (000)	1016.0	2600.0
Crystal size (mm^3)	0.25 × 0.13 × 0.12	0.22 × 0.14 × 0.13
Radiation	CuKα (λ = 1.54184)	CuKα (λ = 1.54184)
2θ range for data collection (°)	6.214–134.102	7.538–134.158

Index ranges	$-3 \leq h \leq 7$, $-21 \leq k \leq 23$, $-22 \leq l \leq 24$	$-13 \leq h \leq 5$, $-14 \leq k \leq 10$, $-50 \leq l \leq 46$
Reflns collected	8923	19686
Independent reflections	4404 [R_{int} = 0.0357, R_{sigma} = 0.0470]	10028 [R_{int} = 0.0615, R_{sigma} = 0.0817]
Data/restraints/parameters	4404/0/318	10028/0/751
GOF on F^2	1.044	1.029
R_1^a, wR_2^b (I>=2σ (I))	0.0473, 0.1211	0.0582, 0.1423
R_1^a, wR_2^b (all data)	0.0510, 0.1256	0.0633, 0.1459
Largest diff. peak/hole / e $Å^{-3}$	0.90/−0.46	0.60/−0.57
Flack parameter	0.000(14)	−0.07(2)

^{a} $R_1 = \Sigma||F^o| - |Fc||/\Sigma F^o|$.

^{b} $wR_2 = [\Sigma w (Fo^2 - Fc^2)^2/\Sigma w (Fo^2)]^{1/2}$.

Table S2. Selected bond distances (Å) and angles (◦) for **HL₄**.

S1-C23	1.683(3)	O1-C3	1.370(5)	O1-C7	1.427(5)
O2-C2	1.344(4)	N1-C8	1.273(5)	N1-C9	1.474(4)
N2-C16	1.457(4)	N2-C23	1.344(4)	N3-C23	1.361(5)
N3-C24	1.426(4)				
C3-O1-C7	117.0(3)	C8-N1-C9	120.7(3)	C23-N2-C16	124.3(3)
C23-N3-C24	126.2(3)	O2-C2-C1	122.0(3)	O2-C2-C3	118.9(3)
O1-C3-C2	115.0(3)	O1-C3-C4	125.2(3)	N1-C8-C1	121.0(3)

N1-C9-C10	109.6(3)	N1-C9-C16	106.2(3)	N2-C16-C9	107.4(3)
N2-C16-C17	111.9(3)	N2-C23-S1	123.4(3)	N2-C23-N3	113.8(3)
N3-C23-S1	122.7(3)				

Table S3. Selected bond distances (Å) and angles (◦) for complexe **L_4**-$ZnNO_3$.

Zn1-S1	2.3135(16)	Zn1-O1	1.946(5)	Zn1-O3	2.043(5)
Zn1-N3	2.020(5)	S1-C11	1.724(7)	O1-C2	1.309(8)
O2-C1	1.370(9)	O2-C7	1.440(9)	O3-N4	1.289(8)
O4-N4	1.239(9)	O5-N4	1.220(9)	N1-C11	1.338(9)
N1-C24	1.421(9)	N2-C10	1.462(9)	N2-C11	1.323(9)
N3-C8	1.292(9)	N3-C9	1.478(9)		
O1-Zn1-S1	125.74(15)	O1-Zn1-O3	104.2(2)	O1-Zn1-N3	97.8(2)
N3-Zn1-S1	113.51(15)	O3-Zn1-S1	106.38(16)	N3-Zn1-O3	107.8(2)
C11-S1-Zn1	104.6(2)	C2- O1- Zn1	122.4(4)	N4-O3-Zn1	109.9(4)
C11-N2-C10	126.2(6)	C8-N3-Zn1	118.5(5)	C9-N3-Zn1	123.8(4)
O4-N4-O3	118.5(6)	O5- N4- O3	118.3(6)	O5-N4-O4	123.2(6)
O1-C2-C3	127.1(6)	C2- C3- C8	124.4(6)	N3-C8-C3	129.2(7)
N3-C9-C10	108.4(5)	N2-C10-C9	111.9(5)	N2-C11-S1	123.7(5)

Section S4 Examples for detection of Zn^{2+} and phosphate anions by sensors

Table S4. Examples for detection of Zn^{2+} and phosphate anions by sensors.

No	Sensor	Detection limit (M)	Identification substance	Reference
1	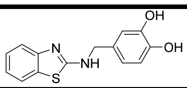	8.21×10^{-8}	Zn^{2+}	40
2	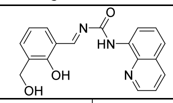	1.0×10^{-7}	Zn^{2+}	41
3	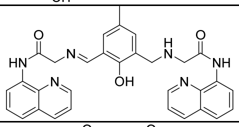	1.0×10^{-7}	Zn^{2+}	42
4	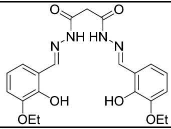	3.2×10^{-6}	$H_2PO_4^-$	7
5	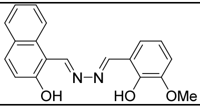	1.0×10^{-4}	Zn^{2+}	43
6	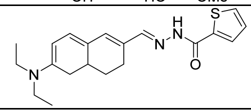	1.0×10^{-4}	Zn^{2+}	44
7	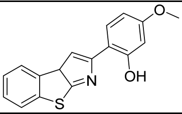	17.8×10^{-3}	Zn^{2+}	45
8	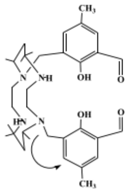	2.7×10^{-11}	Zn^{2+}	46
9	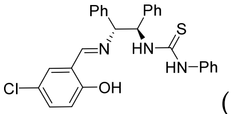 (**HL**$_5$)	2.3×10^{-8}	Zn^{2+}, $H_2PO_4^-$	This work

Section S5 ^{1}H NMR and ^{13}C NMR spectra of **HL₁**–**HL₆** and **3**

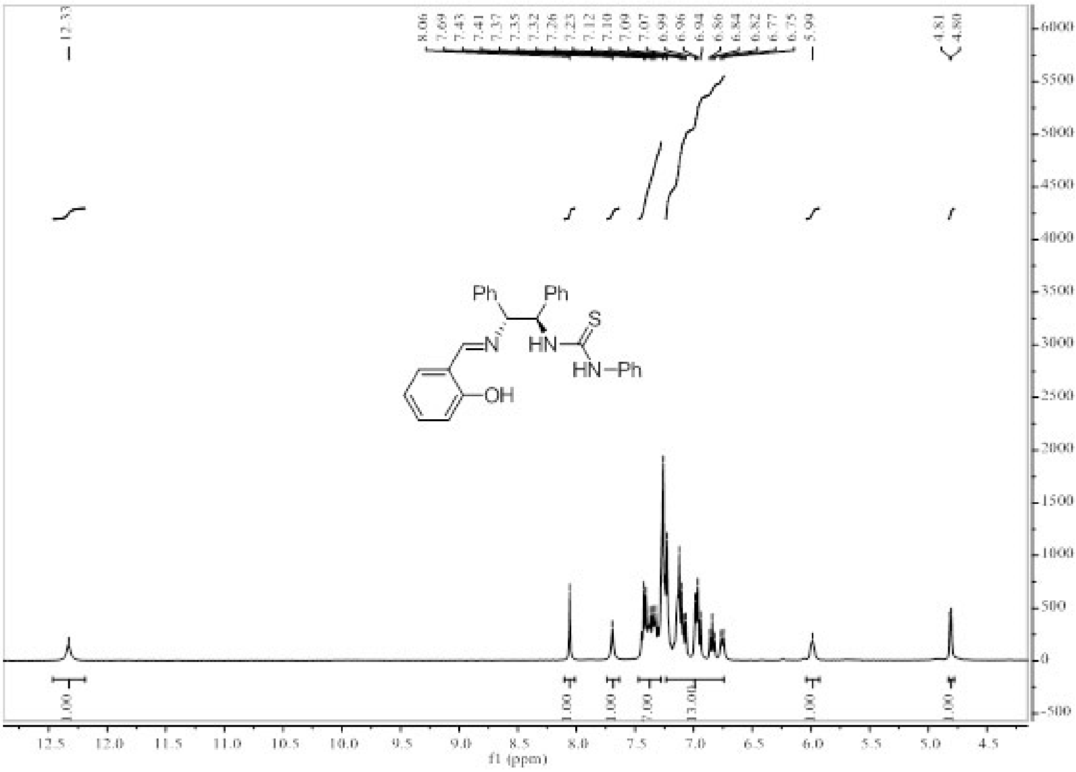

Figure S9. ^{1}H NMR spectra of **HL$_1$**.

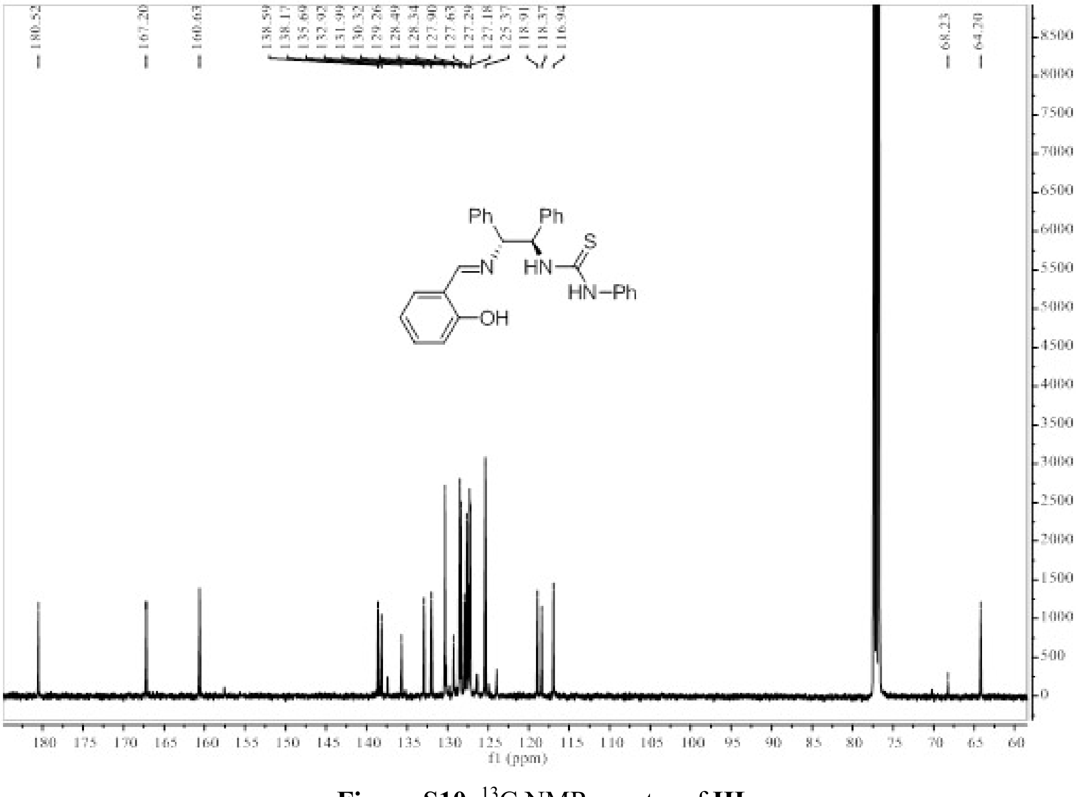

Figure S10. ^{13}C NMR spectra of **HL$_1$**.

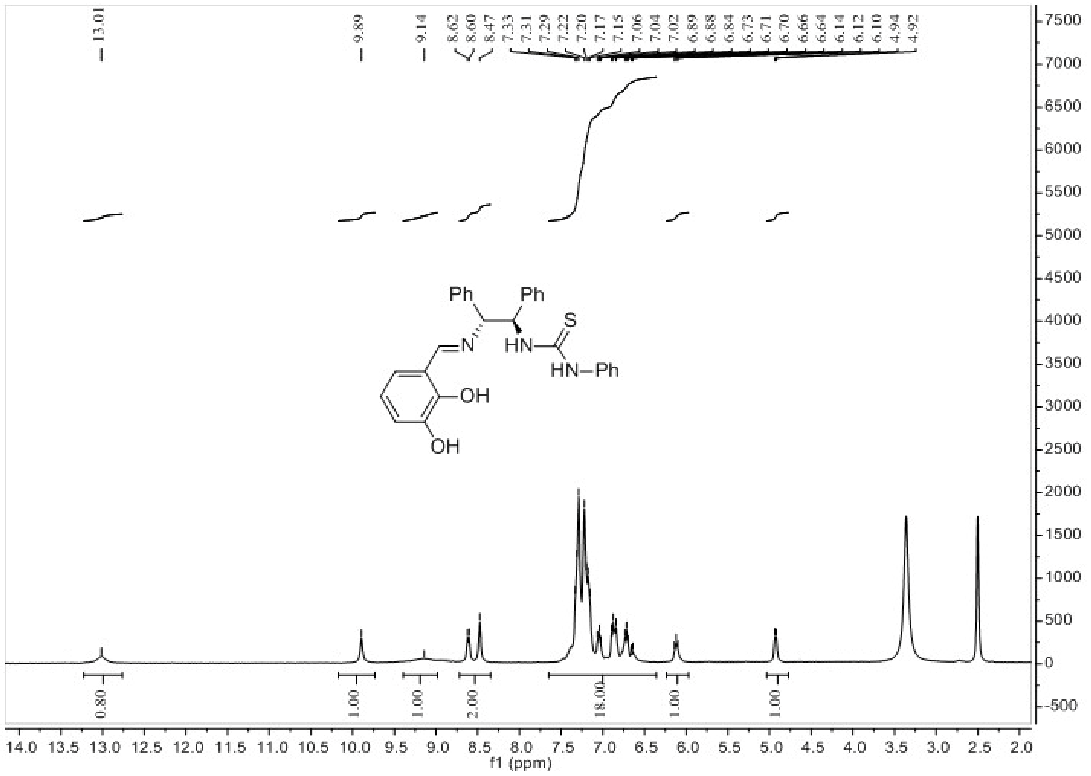

Figure S11. ^{1}H NMR spectra of **HL₂**.

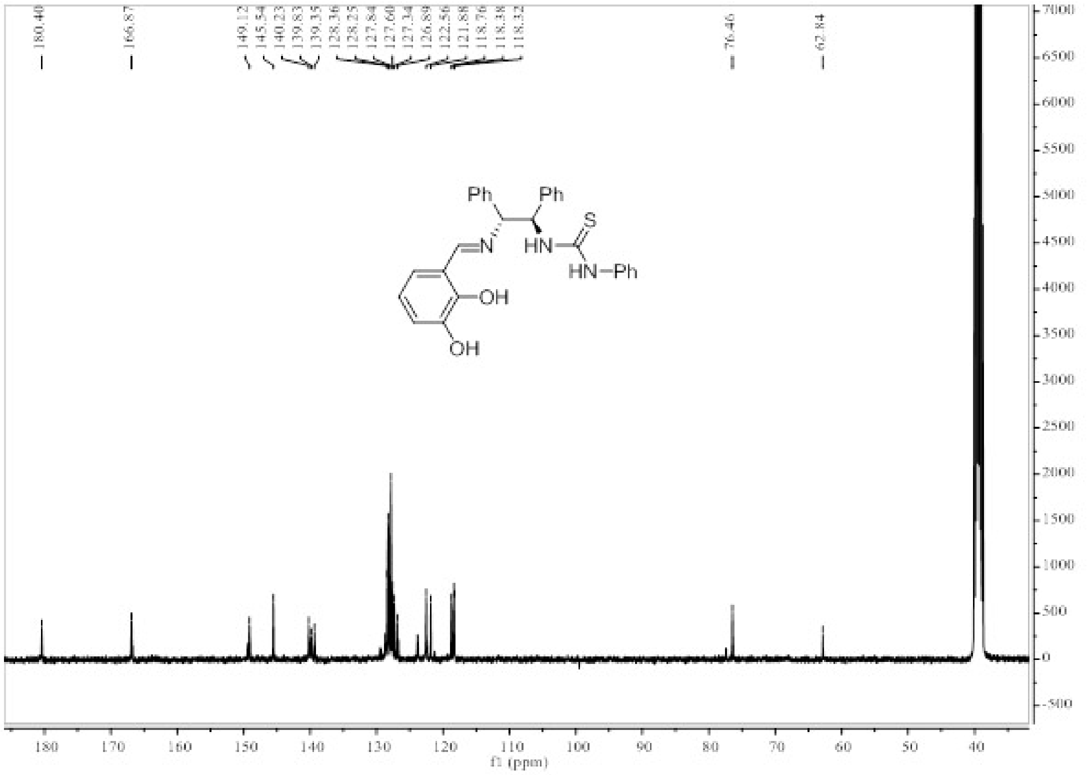

Figure S12. ^{13}C NMR spectra of **HL₂**.

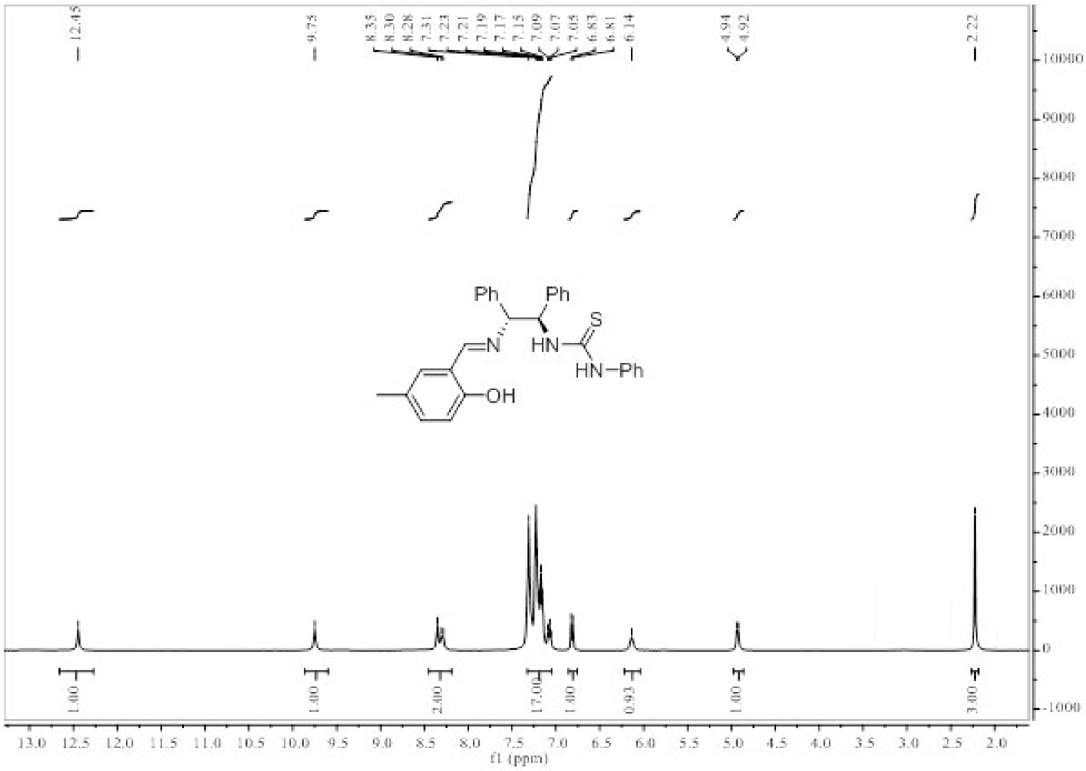

Figure S13. ^{1}H NMR spectra of **HL_3**.

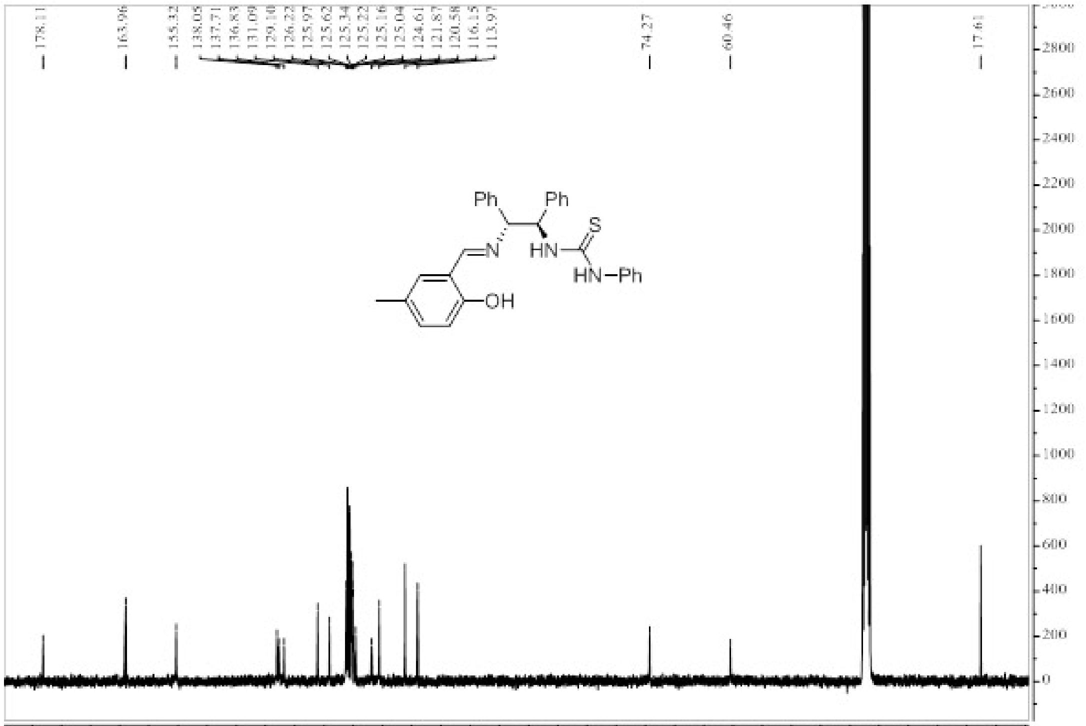

Figure S14. ^{13}C NMR spectra of **HL_3**.

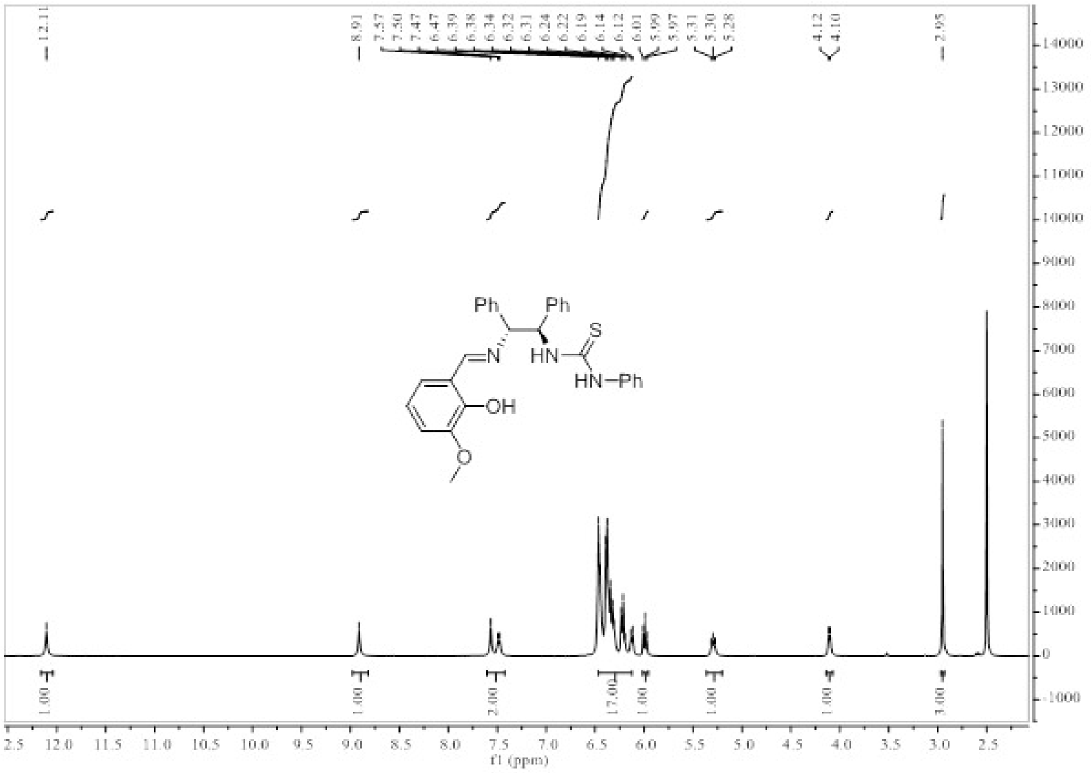

Figure S15. ^{1}H NMR spectra of **HL$_4$**.

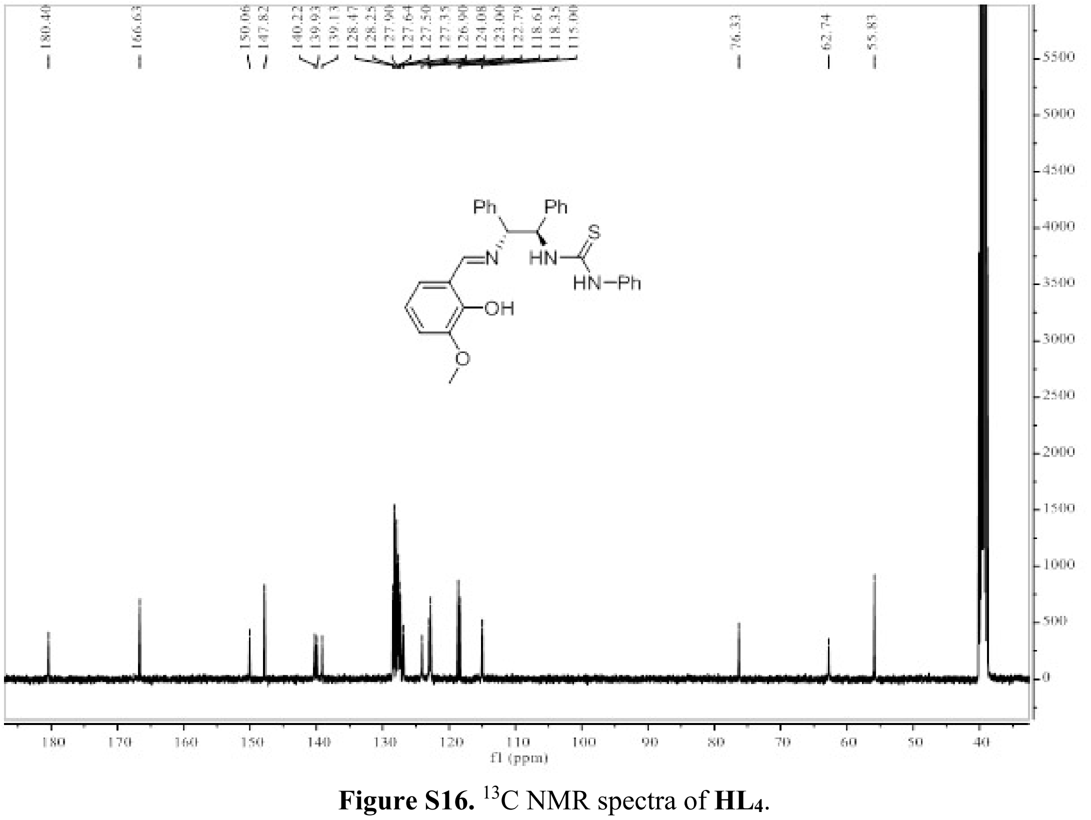

Figure S16. ^{13}C NMR spectra of **HL$_4$**.

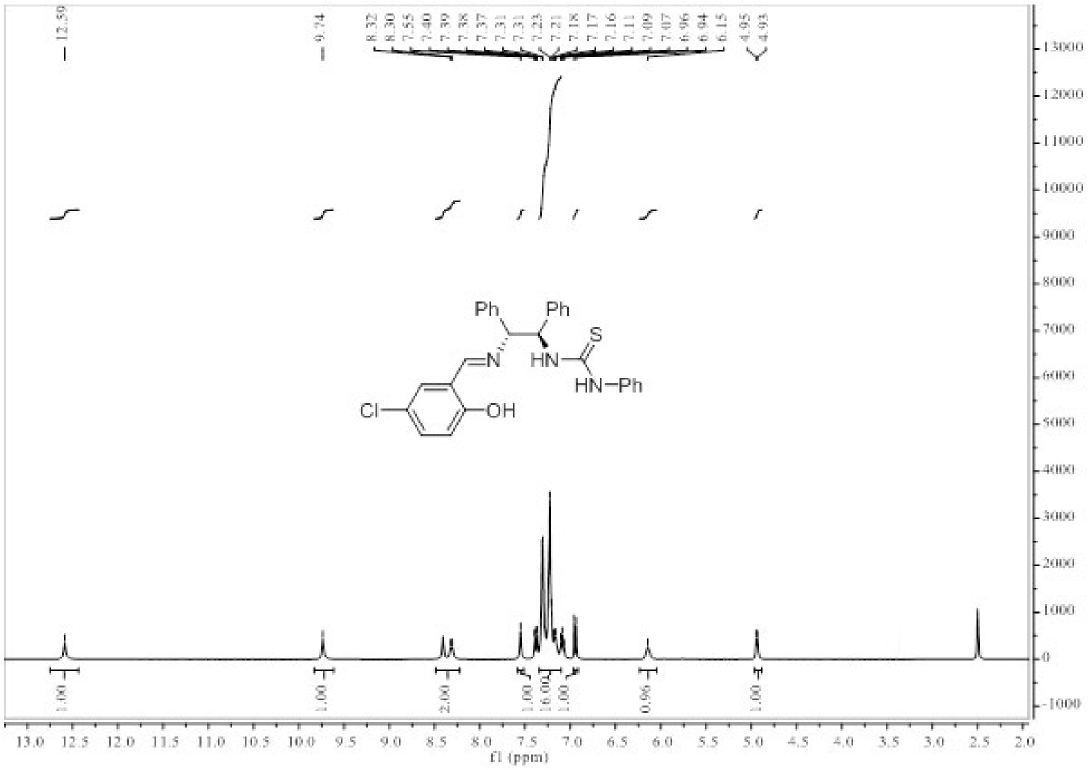

Figure S17. ^{1}H NMR spectra of **HL$_5$**.

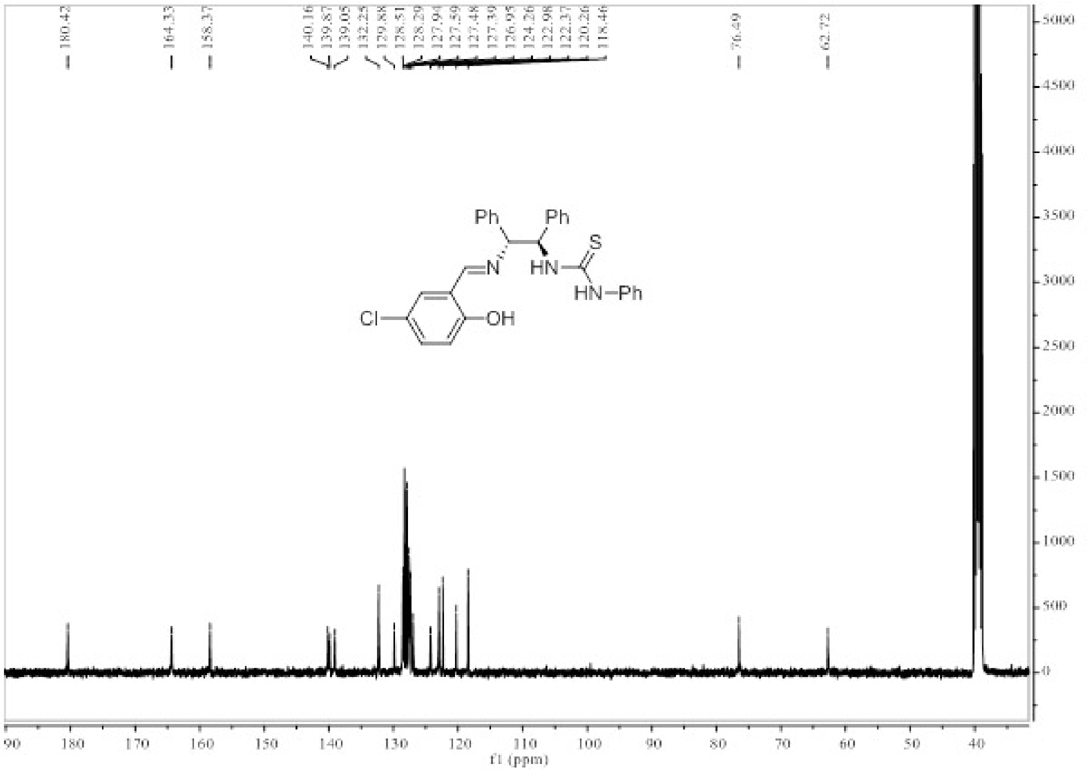

Figure S18. ^{13}C NMR spectra of **HL$_5$**.

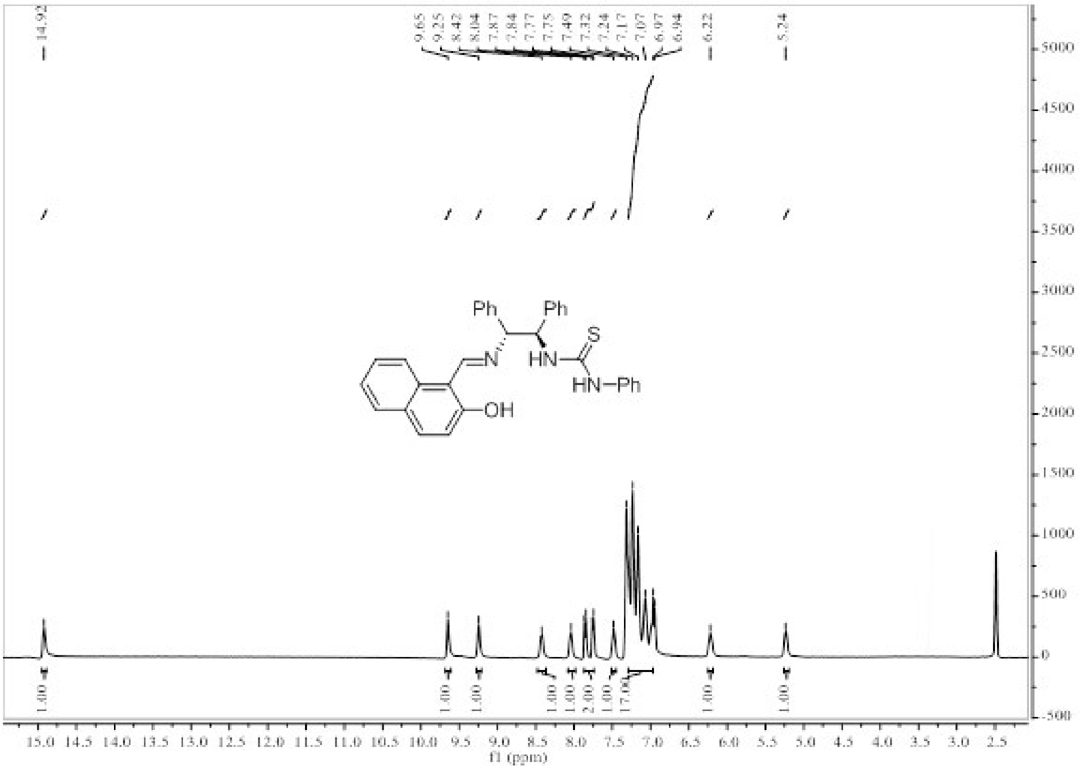

Figure S19. ^{1}H NMR spectra of $\mathbf{HL_6}$.

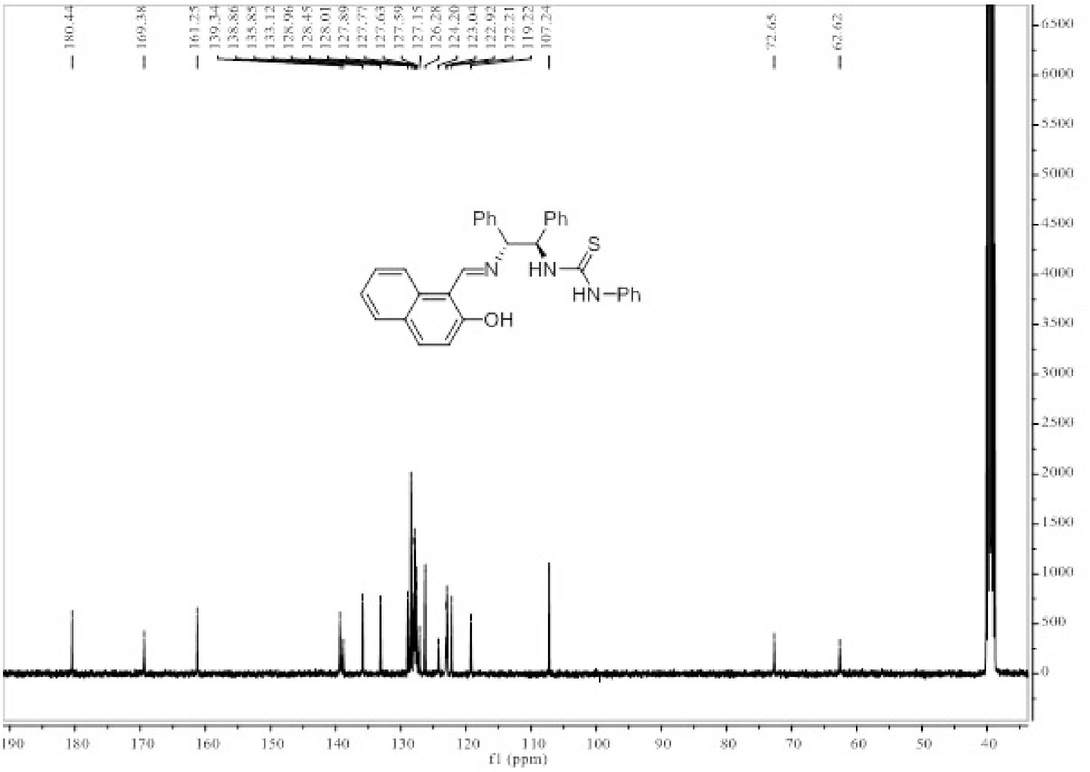

Figure S20. ^{13}C NMR spectra of $\mathbf{HL_6}$.

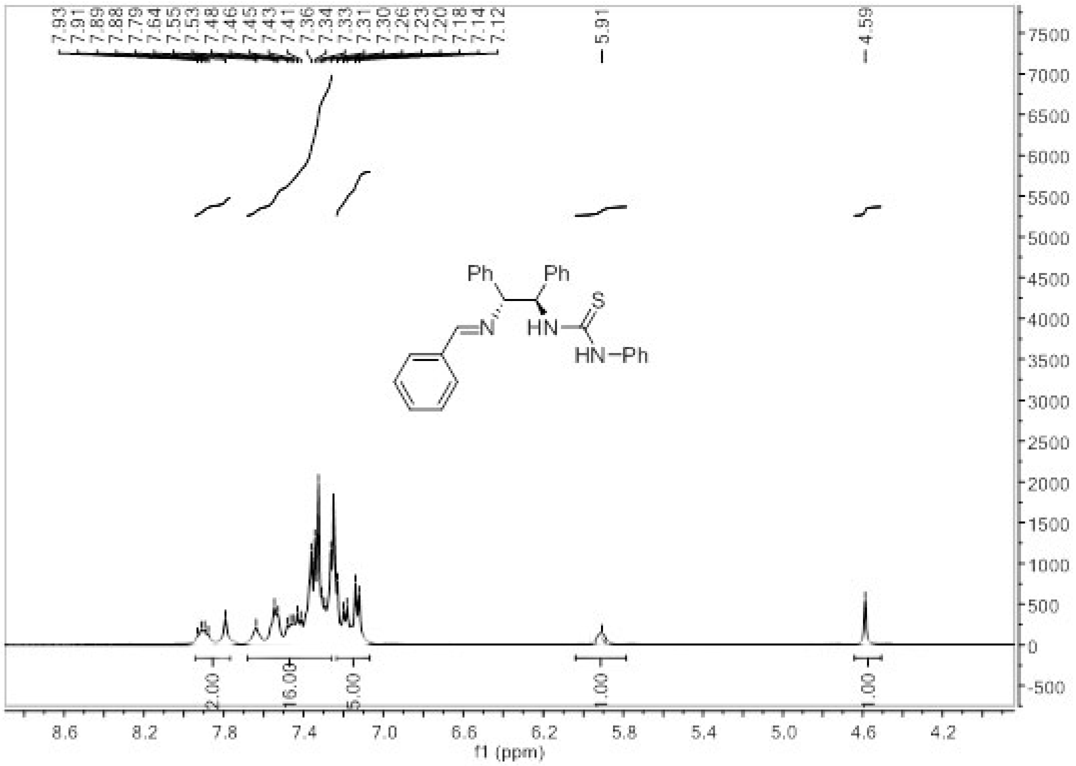

Figure S21. ^{1}H NMR spectra of **3**.

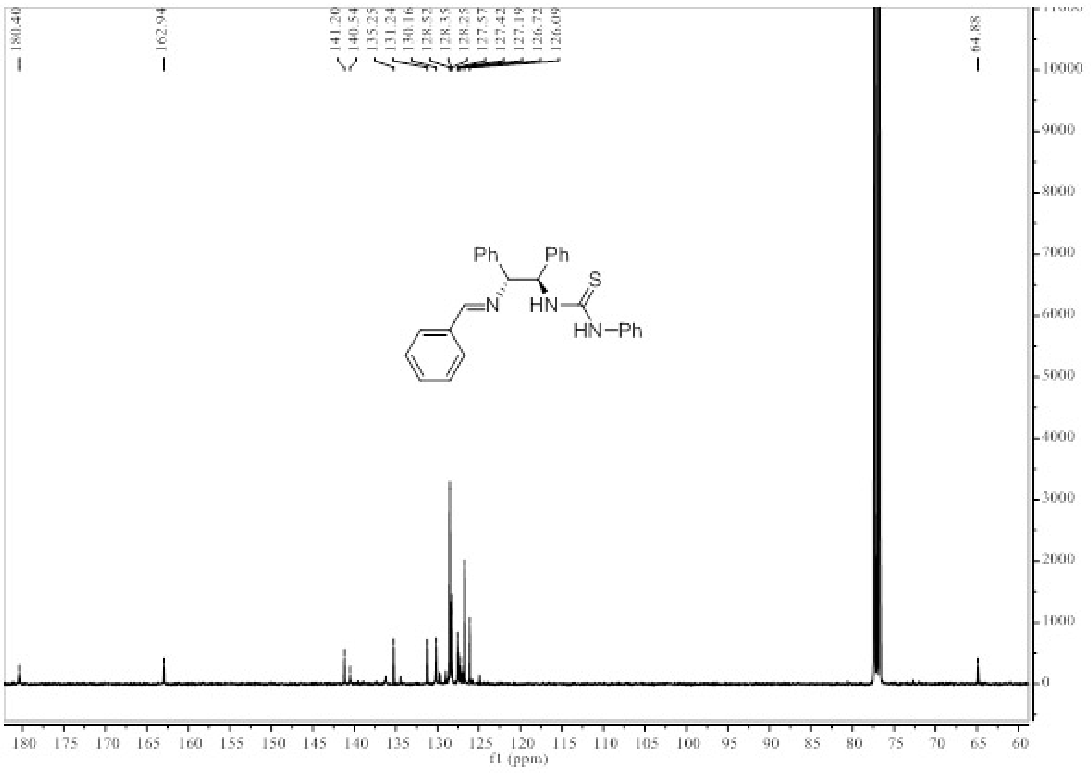

Figure S22. ^{13}C NMR spectra of **3**.
